# Glucose amendments are associated with structural and hormonal shifts in a fungal-algal-bacterial (FAB) consortium

**DOI:** 10.1093/femsmc/xtag051

**Published:** 2026-10-07

**Authors:** Emma Kaszecki, R J Neil Emery, Barry J Saville

**Affiliations:** Environmental and Life Sciences Graduate Program, Trent University, Peterborough, ON K9L 0G2, Canada; Environmental and Life Sciences Graduate Program, Trent University, Peterborough, ON K9L 0G2, Canada; Department of Biology, Trent University, Peterborough, ON K9L 0G2, Canada; Environmental and Life Sciences Graduate Program, Trent University, Peterborough, ON K9L 0G2, Canada; Forensic Science Department, Trent University, Peterborough, ON K9L 0G2, Canada

**Keywords:** bioremediation, symbiosis, *Euglena*, stress response, hormones, microscopy

## Abstract

The fungal-algal-bacterial (FAB) consortium composed of *Euglena mutabilis, Talaromyces*, and *Acidiphilium acidophilum* is a natural acid-tolerant assemblage whose heavy metal handling may depend on interspecies cooperation rather than a single-organism uptake alone. Here, glucose was used as a simplified laboratory model of labile carbon availability to test whether carbon input reorganizes consortium structure and stress responses under CdCl_2_ exposure. Glucose supplementation promoted fungal hyphal development, more extensive visible bacterial colonization, pronounced flocculation, and higher *Euglena* cell densities under CdCl_2_ stress. CdCl_2_ accumulation relative to *Euglena* cell abundance increased over time in all CdCl_2_-treated cultures but did not differ significantly among glucose treatments. Hormone profiling revealed distinct extracellular responses under glucose + CdCl_2_, including elevated bioactive *trans*-Zeatin (*t*Z), gibberellin GA_7_, methyl-thiolated cytokinins, and jasmonic acid, consistent with coordinated consortium-level signalling under metal stress. Scanning electron microscopy provided visual evidence of the FAB physically restructuring when grown in glucose, while transmission electron microscopy (TEM) identified previously uncharacterized electron-dense intravacuole bodies within *E. mutabilis*, although their identity could not be determined. Together, these findings show that glucose availability reorganizes structural and biochemical signalling of the FAB consortium and is associated with changes in consortium organization and physiological responses under heavy metal stress. They also highlight coordinated structural and biochemical responses associated with consortium persistence under acidic, metal-rich conditions and identify directions for future mechanistic investigations.

## Teaser

Glucose addition restructures the fungal-algal-bacterial consortium and boosts coordinated hormone production, cell organization, thereby enhancing community-wide tolerance and growth under heavy metal stress and informing design of engineered bioremediation consortia.

## Introduction

Canada is recognized as a leader in the global mining sector (Natural Resources Canada 2018). Despite its economic significance, the Canadian mining industry produces over one hundred million metric tonnes of solid waste each year (Statistics Canada 2012), which even the most robust regulatory frameworks and engineering interventions cannot guarantee environmental or human safety (Natural Resources Canada 2013, Beckett and Keeling 2019, Crown-Indigenous Relations and Northern Affairs Canada 2022). Traditional restoration practices such as backfilling, adsorption, and permeable reactive barriers can be expensive and inefficient (Moodley et al. 2018, Sun et al. 2018), contributing to the proliferation of tailings.

Bioremediation offers a cost-effective and environmentally sustainable means of reducing the bioavailability by using living organisms to immobilize, transform, or remove contaminants (Jayapal et al. 2023). Although many studies have focused on optimizing single organisms or engineered co-cultures for remediation (Deng and Jia 2011, Babu et al. 2014, Ameen et al. 2020, Brar et al. 2022), naturally occurring microbial consortia from contaminated environments may offer distinct advantages because their members are already adapted to acidic, metal-rich conditions and have evolved cooperative interactions that enhance survival (Chen et al. 2018, Wang et al. 2021, Zhang et al. 2022, Rasheed et al. 2023, Abbasi et al. 2024, Sonker et al. 2024).

Among acid-tolerant protists, *Euglena mutabilis* is particularly well suited to contaminated environments because of its metabolic flexibility and tolerance to heavy metals (Kostygov et al. 2021, Ebenezer et al. 2022). These characteristics make it a promising member of natural bioremediation consortia, capable of maintaining biologically active biomass under harsh conditions.

Heavy metal retention in microbial consortia arises through complementary mechanisms, including surface adsorption, intracellular sequestration, biomass flocculation, and metabolic cooperation among community members (Nakatsu and Hutchinson 1988, Das et al. 2009, Chandrashekharaiah et al. 2022). In addition to these physical processes, signalling molecules such as auxins, cytokinins (CKs), jasmonates, and gibberellins may coordinate growth and stress responses among microbial partners (Dao et al. 2020, Aloo et al. 2023, Nguyen et al. 2023, Lin et al. 2025). Hormone profiling therefore provides a means of investigating consortium-level responses to heavy metal stress.

Recent studies revealed that *E. mutabilis* is more tolerant to heavy metals, including cadmium, than other euglenoid species (Kennedy et al. 2024). When isolated from these extreme environments, *E. mutabilis* is frequently associated with other organisms, such as the extremophilic bacterium *Acidiphilium acidophilum* and metal-tolerant fungi *Talaromyces* (Nakatsu and Hutchinson 1988). Within this consortium, the three partners may play complementary roles. *Euglena mutabilis* provides an acidic- and metal-tolerant, metabolically flexible host capable of maintaining biomass under harsh conditions (Kaszecki et al. 2026). *Talaromyces* may contribute extracellular structure, aggregation, and flocculation, as well as metabolites and hormones that influence community growth and stress tolerance (Hussain et al. 2025). *Acidiphilium*, as an acidophilic heterotroph recurrently found in acidic mine waters, may support consortium persistence through metabolic exchange and iron-transforming activities common among *Acidiphilium* spp. under low-pH conditions (Johnson and Bridge 2002, Coupland and Johnson 2008). Together, these interactions may improve survival and maintain biologically active biomass under conditions relevant to mine tailings remediation.

Carbon availability may further influence these interactions by altering the relative activity of consortium members. Among the diverse dissolved organic carbon compounds present in acid mine drainage, glucose was selected as a simplified laboratory model of readily bioavailable carbon because it provides a reproducible substrate for experimentally manipulating carbon availability and evaluating microbial community responses (Ghosh and Leff 2013, She et al. 2022). Regulation of carbon availability is widely used in microalgal-fungal systems to influence biomass production, community interactions, and resource recovery, with glucose representing one of the principal organic carbon sources used to investigate these processes under controlled laboratory conditions (Wu et al. 2024). In this study, glucose was used as a simplified model of labile carbon availability to determine whether heterotrophic carbon enhances consortium performance during CdCl_2_ stress. Although *E. mutabilis* has been described as an obligate photoautotroph under axenic conditions (Olaveson and Stokes 1989), glucose may stimulate fungal and bacterial partners, thereby altering consortium structure and stress response.

This study investigated how glucose availability alters the physiology, structure, metal accumulation, and hormone dynamics of a naturally occurring fungal-algal-bacterial (FAB) consortium containing *E. mutabilis* under CdCl_2_ stress. By integrating growth measurements, microscopy, ICP-MS, and hormone profiling, we sought to characterize the coordinated physiological and biochemical responses associated with consortium resilience in acidic, metal-rich envrionments and evaluate their relevance to bioremediation.

## Methods and materials

### Culture selection and growth conditions of *E. mutabilis*

Field samples of *E. mutabilis* isolated from a gold mine were obtained from the Canadian Phycological Culture Centre (CPCC, University of Waterloo, Canada). The strain (CPCC 657) has been maintained in laboratory culture for several years as a stable, naturally occurring non-axenic consortium comprising *E. mutabilis*, a fungus from the genus *Talaromyces*, and the bacterium *Acidiphilium acidophilum*. Previous molecular characterization assigned the fungal isolate to the genus *Talaromyces*, but species-level identification could not be confirmed (Kaszecki et al. 2024); therefore, it is referred to as *Talaromyces* sp. throughout the study. Although this community may not reflect the original field assemblage, it provides a reproducible model for studying microbial interactions under defined laboratory conditions. As previously reported, the *E. mutabilis* strain used in this study cannot be maintained axenically without loss of viability (Kaszecki et al. 2024). Isolation of the associated fungus and bacterium was beyond the scope of the present study. The objective was to characterize physiological and biochemical responses of the naturally maintained consortium to examine consortium-level responses rather than those of isolated members. Therefore, all experiments were conducted on the intact consortium to preserve the natural interactions and characteristics of this system.

Stock cultures were grown in modified acid medium (MAM) (Olaveson and Stokes 1989) with modifications recommended by the CPCC, including adjusting to a pH of 4.3 (Canadian Phycological Culture Centre 2017). pH was not treated as an experimental variable in this study. Filter sterilized F/2 nutrient solution was added to autoclaved medium after it cooled. Cultures were grown autotrophically in 250 ml Pyrex Erlenmeyer flasks secured with foam stoppers covered in aluminum foil under standard aeration (100 RPM on a Thermo Fisher Scientific MaxQ 3000 shaker) while cycling light and temperature (16:8 LD cycle at 260 µm s^−1^ m^−1^; 24°C ± 0.5°C in light and 18°C ± 0.5°C in dark) in a Conviron PCG20 environmental chamber.

Preliminary screening experiments were conducted to select suitable glucose and CdCl_2_ concentrations for subsequent experiments. Serial dilutions of glucose (20, 10, 5, and 2.5 g/l) were prepared using MAM medium and tested. Glucose treatments were evaluated over 120 h by measuring total chlorophyll content (Table S1). Chlorophyll responses were analysed independently at each sampling time using one-way ANOVA followed by Tukey’s HSD test. The purpose of this screening was to confirm that glucose supplementation elicited measurable physiological responses across the tested concentration range and to identify experimentally tractable treatment concentrations. Glucose concentrations above 5 g/l consistently produced excessive flocculation that interfered with reproducible sampling and quantitative enumeration of *E. mutabilis*. Lower glucose concentrations were not selected because the objective was to evaluate the highest glucose amendments that could be reliably analysed while avoiding the analytical limitations associated with excessive floccuation. Consequently, 2.5 and 5 g/l were selected as the highest experimentally tractable glucose concentrations for the main study. A CdCl_2_ concentration of 100 µM was selected because it represents a sublethal, environmentally relevant level comparable to those reported in acid mine drainage systems (Olaveson and Nalewajko 2000) and has previously been shown to elicit a measurable physiological response in the FAB consortium (Kaszecki et al. 2024).

Cell counts of *E. mutabilis* from stock cultures, also grown in MAM, were performed using a hemocytometer (Hausser Scientific). A volume of cells from each stock containing 12 × 10^6^ cells was aliquoted into each 15 ml Falcon tube. Tubes were centrifuged (3 724 RCF for 10 min) and the supernatant was discarded. The pellet was inoculated into 250 ml Pyrex Erlenmeyer flasks containing 60 ml of media for a final concentration of 200 000 *E. mutabilis* cells/ml. Control cultures were grown in MAM, MAM with 5 g/l of glucose (Fisher Scientific Cat. No. D16-1) (MAM + 5), or MAM with 2.5 g/l of glucose (MAM + 2.5) through serial dilutions. Furthermore, cultures were grown in the same three medium conditions with the addition of 100 µM CdCl_2_. Each experimental condition was carried out in triplicate.

Flasks were placed in the Conviron PCG20 environmental chamber under the same light and temperature conditions as the stock cultures for 5 days, corresponding with the duration of time used to assess physiological and biochemical effects of heavy metals on microalgae in previous studies (Naorbe and Serano 2018, Hussain et al. 2020, Wang et al. 2022). During the experiment, the flasks were agitated for 1 min every 24 h (80 RPM on a Thermo Fisher Scientific MaxQ 3000) but were otherwise unshaken. *Euglena mutabilis* cell counts were assessed 1- (24 h), 3- (72 h), and 5 days (120 h) post-inoculation. To reduce aggregation prior to counting, cultures were gently mixed and diluted 1:10 in sterile dH_2_O, which partially dispersed flocs and allowed visualization of individual cells. Counts were made under light microscopy at 10x magnification based on the distinctive morphology and motility of *E. mutabilis*, which could be clearly differentiated from the filamentous *Talaromyces* hyphae and small, non-motile *Acidiphilium* cells. Five technical counts were performed for each replicate to minimize variability. Because aggregation may influence cell accessibility, we interpreted cell counts alongside chlorophyll content (2.3) and CFU recovery (2.3). Similar challenges associated with quantifying cells in flocculated algae have been previously recognized (Pell et al. 2017), with dilution of flocculation used as a strategy to improve cell enumeration in other systems (Calleja and Johnson 1977).

Additionally, aliquots were obtained to quantify cellular CdCl_2_ accumulation and hormone profiles 1, 3, and 5 days post-inoculation. Cultures were also visualized using a scanning electron microscope (SEM) after 5 days of growth.

### *Euglena* cell viability after CdCl_2_ exposure

At 1, 3, and 5 days post-inoculation, 100 µl aliquots were taken from each flask and diluted with 400 µl of a 0.4% Trypan Blue solution (Bioshop Cat. No. TRY477.100) in PBS (Bioshop, Cat. No. PBS404.100). Cell viability of *E. mutabilis* was assessed by visualizing uptake of the Trypan Blue stain and counting the number of living and dead cells using a hemocytometer (Hausser Scientific).

### Assessment of post-stress growth

Recovery of *E. mutabilis* and *Talaromyces* was assessed by plating on potato dextrose agar (PDA) plates prepared using a 1.4% (w/v) agar mixture which was sterilized by autoclaving. A total of 15 ml of media was added to each plate (Greiner Bio-One Cat. No. 633 181). A 100 µl aliquot from each flask was diluted with 900 µl of sterile 0.9% (w/v) NaCl and 50 µl was added to an agar plate. Plates were sealed with parafilm and incubated at 22°C in darkness. The number of *E. mutabilis* and *Talaromyces* colony forming units (CFUs) were recorded after 1 and 7 days of growth. Because fungal colonies frequently formed confluent lawns under glucose supplementation, CFUs are presented as semi-quantitative indicators of post-stress recovery and viability rather than absolute measures of fungal biomass.

### Assessment of chlorophyll content

Chlorophyll content was assessed according to Kaszecki et al. (2024). Briefly, 500 µl from each flask was aliquoted into 2 ml Eppendorf tubes containing glass beads (Sigma Aldrich Cat. No. G8772) and the media was decanted after centrifugation at 4°C (21 130 RCF for 2 min). Total 1 ml of chilled methanol (MeOH) was added to each tube to extract the chlorophyll, and the samples were agitated at 30 Hz for 2 min (Retsch Mixer Mill MM 400). After centrifugation to pellet the glass beads (21 130 RCF for 2 min), the MeOH was transferred to a fresh Eppendorf tube and the extraction was repeated. Total 200 µl of extracted MeOH was added to three 96-well optic plates (ThermoFisher Scientific Cat. No. 165 305) for triplicate readings on a BioTek Synergy HTX Multimode Reader at 652 and 665 nm wavelengths. Chlorophyll content was quantified according to Warren (2008) and normalized against *E. mutabilis* cell counts.

### Cellular CdCl_2_ accumulation

Samples were extracted according to Metcalfe et al. (2018) with modifications. Total of 1 ml from each flask was added to a 1.5 ml microfuge tube. The supernatant was decanted with a pipette following centrifugation (3 824 RCF for 10 min). Cell pellets were washed three times by resuspending in 2 ml of sterile dH_2_O and decanting through centrifugation. Since mild washing may not completely eliminate surface-adsorbed CdCl_2_, measured concentrations represent total cell-associated CdCl_2–_both intracellular and surface-bound. After the final wash, 300 µl of 70% (v/v) nitric acid (HNO_3_) (Fisher Scientific Cat. No. A509P500) was added and each tube was placed on a heat block at 95°C for 20 min to digest the pellet. The entire volume of digested sample was diluted in 10.2 ml of sterile dH_2_O for a final HNO_3_ concentration of 2% (v/v) and filtered through a 0.45 µm polytetrafluoroethylene (PTFE) syringe filter (VWR Cat. No. 76479–004) into a 15 ml Falcon tube. Media blanks for each condition were extracted in parallel. Corresponding supernatant concentrations of CdCl_2_ were not assessed.

Elemental analysis was conducted using an Agilent 8900 ICP-QQQ-MS in the Water Quality Centre (Trent University). Sample introduction was executed using a MicroMist nebulizer (nominal uptake rate: 400 µl min⁻¹) coupled with a Scott double-pass spray chamber to ensure efficient aerosol formation. The instrument was operated with a radio frequency (RF) power of 1550 W and a carrier gas flow rate of 1.05 l min⁻¹, following the General-Purpose Plasma Preset parameters. The sampling depth was set to 10 mm, and an X-type extraction lens was used to optimize ion transmission. Sample introduction into the plasma was facilitated by standard nickel sampler and skimmer cones. Cadmium (Cd) was analyzed in single quadrupole mode using helium (He) as the collision cell gas. Calibration standards were prepared through serial dilution of 1000 ng ml⁻¹ single-element standards in 2% HNO_3_ (Fig. S1). Rhodium served as the internal standard and was introduced continuously inline throughout the analysis. All solutions were prepared using high-purity water (18.2 MΩ·cm) and ultrapure HNO₃ to ensure minimal contamination.

CdCl_2_ accumulation was subsequently normalized to *E. mutabilis* cell counts to provide a consistent basis for comparing consortium-associated CdCl_2_ retention relative to *E. mutabilis* abundance across treatments. Since fungal and bacterial biomass were not quantified independently, these measurements represent total CdCl_2_ associated with the harvested consortium biomass and do not resolve the relative contribution of individual consortium members to total CdCl_2_ retention.

### Hormone profiling

#### Solid phase extraction and purification of hormones

From each flask, 10 ml of culture was collected into 50 ml Falcon tubes 1, 3- and 5 days post-inoculation. The tubes were centrifuged (3724 RCF for 10 min) to form a cell pellet, and the supernatant was decanted into a new 50 ml Falcon tube. From there, the supernatant was sterilized through a 0.2 µm polyethersulfone (PES) syringe filter (Fisher Scientific Cat. No. 13–1001-06) into a fresh 50 ml Falcon tube. Media blanks were prepared the same way at the same time. The cell pellets were washed by resuspension in sterile 0.9% NaCl, centrifuged, and the supernatant discarded. Prepared samples were flash-frozen with liquid nitrogen (LN_2_), freeze-dried (LabConco Free Zone lyophilizer; Kansas City, MO, USA), and stored at −80°C.

Hormone extractions were performed sequentially to obtain distinct hormone fractions, following modified methods previously published (Šimura et al. 2018, Gibb et al. 2020). This approach enabled the extraction of 39 CKs and acidic hormones—including abscisic acid (ABA), gibberellins (GA_1_, GA_4_, GA_7_, GA_9_, GA_20_), indole-3-acetic acid (IAA), jasmonic acid (JA), and salicylic acid (SA)—from a single sample (Table S2; Method S1).

### Visualization with SEM

Samples were prepared for SEM according to the air-drying technique for cultured micro-organisms outlined by Murtey and Ramasamy (2021), with modifications. Total 1 ml of culture was aliquoted into 1.5 ml microfuge tubes. The supernatant was decanted after centrifugation (3824 RCF for 10 min) and the pellet was resuspended in 500 µl of 2.5% glutaraldehyde (Sigma Aldrich Cat. No. G5882-100ml) prepared in a 0.1 M phosphate buffer (pH 7.2) for 16 h at room temperature. After fixation, the sample was centrifuged (10 621 RCF for 2 min) and the glutaraldehyde was removed. The pellet was resuspended in 1 ml of the 0.1 M phosphate buffer for 10 min, centrifuged to decant, and the wash step was repeated. The pellet was then dehydrated through an EtOH series (35% EtOH in dH_2_O, 50% EtOH in dH_2_O, 75% EtOH in dH_2_O, 95% EtOH in dH_2_O, 2 × 100% EtOH) followed by two washes with HMDS (Sigma Aldrich Cat. No. 440191–100ML). At each step, 200 µl of solvent was added to the tubes for 10 min after which the tubes were centrifuged and the supernatant discarded. After the final HMDS wash, the sample tubes were left open in a fume hood overnight to air dry.

Dehydrated samples were visualized with a Hitachi FlexSEM 1000 II connected to an energy-dispersive X-ray spectrometer (QUANTAX Compact 30 with 129 eV Xflash Detector 630Mini) at 15.0 kV.

### Visualization with TEM

Total 1 ml of cultures grown in MAM and MAM + 100 µM CdCl_2_ were aliquoted into 1.5 ml microfuge tubes. The supernatant was decanted with a p200 following centrifugation, the pellet was resuspended in 500 µl of 2.5% glutaraldehyde (Sigma Aldrich Cat. No. G5882-100ML) prepared in a 0.1 mol/l phosphate buffer (pH 7.2) for 24 h at room temperature. Sample preparation and embedding were performed by the Cellular and Molecular Electron Microscopy core (The Hospital for Sick Children, Toronto, Canada). The fixed tissue was sectioned into 2 mm cubes and rinsed with 0.1 M sodium cacodylate buffer with 0.2 M sucrose (pH 7.3) for 10 min. Samples were post-fixed in 1% osmium tetroxide in the same buffer for 1.5 h, then rinsed again with the sucrose-buffer rinse. Dehydration was performed through a graded EtOH series (70% EtOH in dH_2_O, 90% EtOH in dH_2_O, 3 × 100% EtOH) followed by two washes with propylene oxide. Samples were then infiltrated with a 50:50 mixture of propylene oxide and Spurr resin for 1 h, followed by two washes of pure Spurr resin (1 h and overnight) and then embedded in molds and polymerized at 65 °C overnight. Ultrathin sections (90 nm) were cut on an ultramicrotome using a diamond knife, mounted on copper grids, and contrasted with uranyl acetate and lead citrate before imaging on a Hitachi HT7800.

### Statistical analysis

For each response variable (cell counts, chlorophyll content, and each hormone), differences among treatments were analyzed separately for each sampling day using a one-way ANOVA (*P* < 0.05) followed by Tukey’s Honest Significant Difference (HSD) test. Blank media samples containing each glucose concentration with and without CdCl_2_ were processed and analyzed using the same methods to remove background hormone levels (Table S3). Experiments were performed once in a single batch with three independent biological replicates per treatment and time point. Future work will test reproducibility across independent runs.

## Results and discussion

### FAB growth and chlorophyll content are dependent on the presence of glucose and CdCl_2_

Glucose was used in this study as a simplified model to investigate how labile carbon availability reshapes the physiology and structure of the FAB consortium under CdCl_2_ stress in controlled conditions. After 5 days of growth without standard aeration, the FAB cultures supplemented with glucose contained flocs (Fig. 1). Glucose also promoted more extensive visible fungal hyphal development compared to cultures without glucose. The addition of CdCl_2_ to cultures with glucose supplementation did not alter the level of flocculation and the *E. mutabilis* cells remained embedded in what appears to be a fungal-derived mycelial mat with extracellular material. In contrast, when cultured without glucose supplementation either in the presence or absence of Cd, the *E. mutabilis* cells were either free floating or more loosely associated with the other FAB members and the overall culture had a diffuse green cast that was not present with glucose supplementation (Fig. 1). Additionally, cultures lacking both glucose and CdCl_2_ displayed more limited biomass, and those exposed to CdCl_2_ without glucose showed the most suppressed growth. These observations suggest that glucose availability promoted bioflocculation within the FAB consortium, potentially through the increased fungal proliferation observed in glucose-amended cultures (Fig. 1).

**Figure 1:**
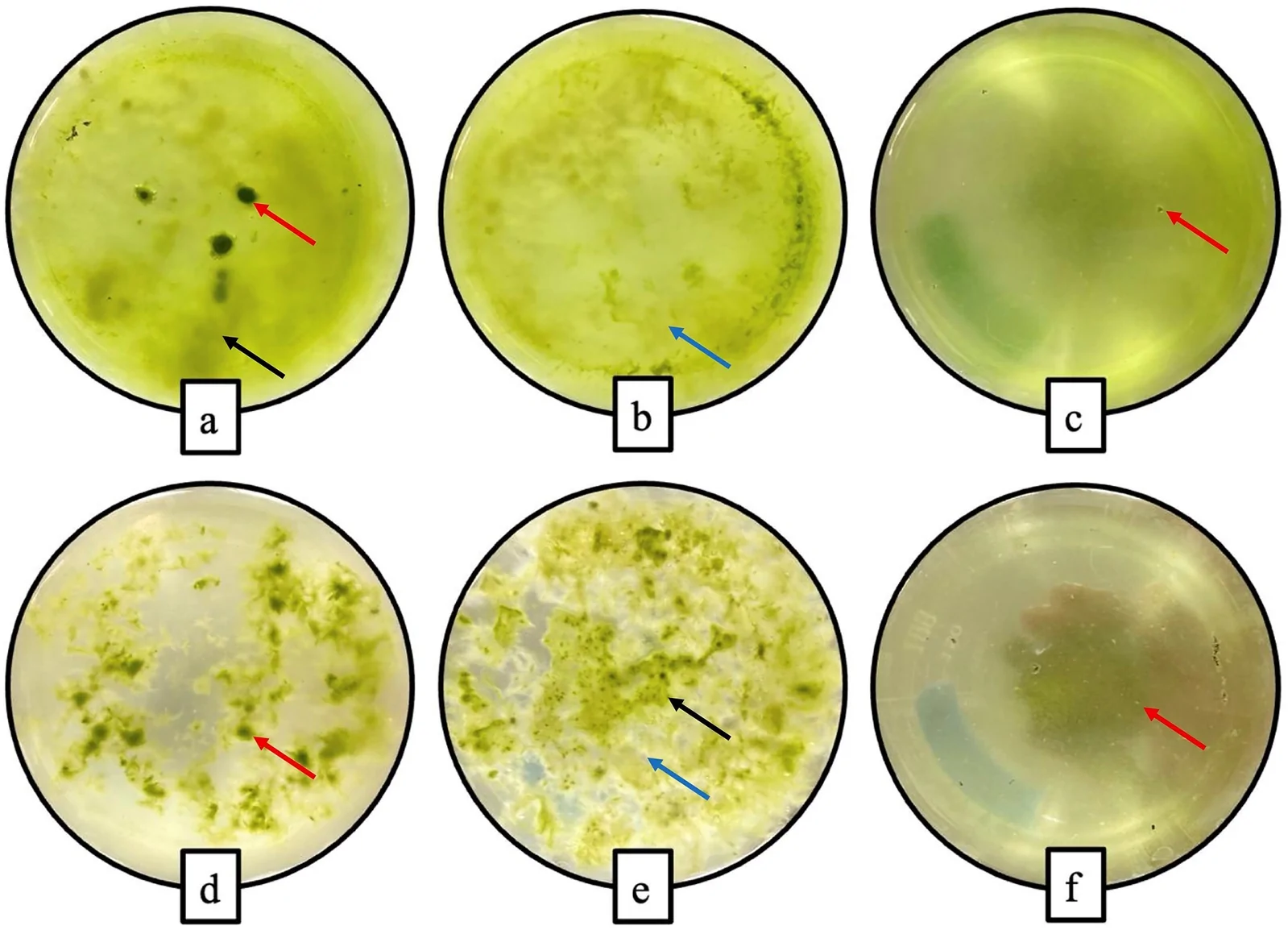
Representative bottom-view images of FAB consortium culture after 5 days of growth under glucose and CdCl_2_ treatments. Cultures were grown in the absence and presence of CdCl_2_ and supplemented with 0, 2.5, or 5 g/l glucose, as indicated in each panel. The untreated control (c; 0 g/l glucose, 0 µM CdCl_2_) illustrates the characteristic morphology of the consortium under standard culture conditions. Red arrows indicate regions of concentrated *E. mutabilis* biomass, blue arrows indicate visible mycelial mats, and black arrows indicate flocculated aggregates. These images are presented as qualitative observations of consortium morphology and are intended to complement, rather than replace, the quantitative physiological and biochemical measurements presented elsewhere.

Bioflocculation technologies are increasingly explored for wastewater treatments, biomass harvesting, and bioremediation (Ramanan et al. 2016, Ayangbenro et al. 2019). Most current bioflocculation systems utilize bacterial biofilms or algal extracellular polymeric substances (EPS) that aggregate particles to facilitate separation of solid and organic matter from liquid phases (Yang et al. 2024a, Kurniawan et al. 2022); however, these often require precise environmental conditions and are limited by low mechanical stability or slow processing rates (Yang et al. 2024b, Manheim and Nelson 2013, Mishra 2016, Matter et al. 2019). To overcome such limitations, co-culturing approaches have been investigated, wherein one organism enhances the flocculating efficiency of another. Filamentous fungi, like *Talaromyces*, offer significant advantages for bioflocculation due to their robust mycelial network, high surface area, and EPS secretion (Muradov et al. 2015, Bhattacharya et al. 2017, Nazari et al. 2020). Pellet-assisted flocculation systems, similar to the morphology observed in this study, use actively growing fungal hyphae that entangle or bind to microalgae, forming dense and stable flocs (Wang et al. 2023). This is especially advantageous in acidic, heavy metal-rich environments. Acidic pH not only supports the growth of these organisms but also enhances pellet formation in fungi, as the lower pH increases the hydrophobicity of fungal hyphae through the protonation of surface functional groups (Chu et al. 2021, Bansfield et al. 2022). Acidification also destabilizes algal cells by reducing their surface charge, thereby promoting close cell-to-cell contact and facilitating effective flocculation (Laezza et al. 2022).

In addition to these structural changes, glucose and CdCl_2_ exposure were associated with differences in *E. mutabilis* abundance within the FAB consortium (Fig. 2). Relative to the untreated control, glucose-supplemented cultures exhibited increased visible mycelial growth and aggregation, whereas CdCl_2_-exposed cultures showed a greater tendency towards flocculated structures. While *E. mutabilis* enumeration provided an index of algal population changes, it is acknowledged that accurate quantification within the mixed consortium remains challenging due to cellular aggregation and the presence of multiple taxa. This limitation is addressed by integrating independent metrics including chlorophyll content (Fig. S2) and CFU counts (Table S4), which together allowed for a more holistic interpretation of FAB growth and CdCl_2_ stress response.

**Figure 2:**
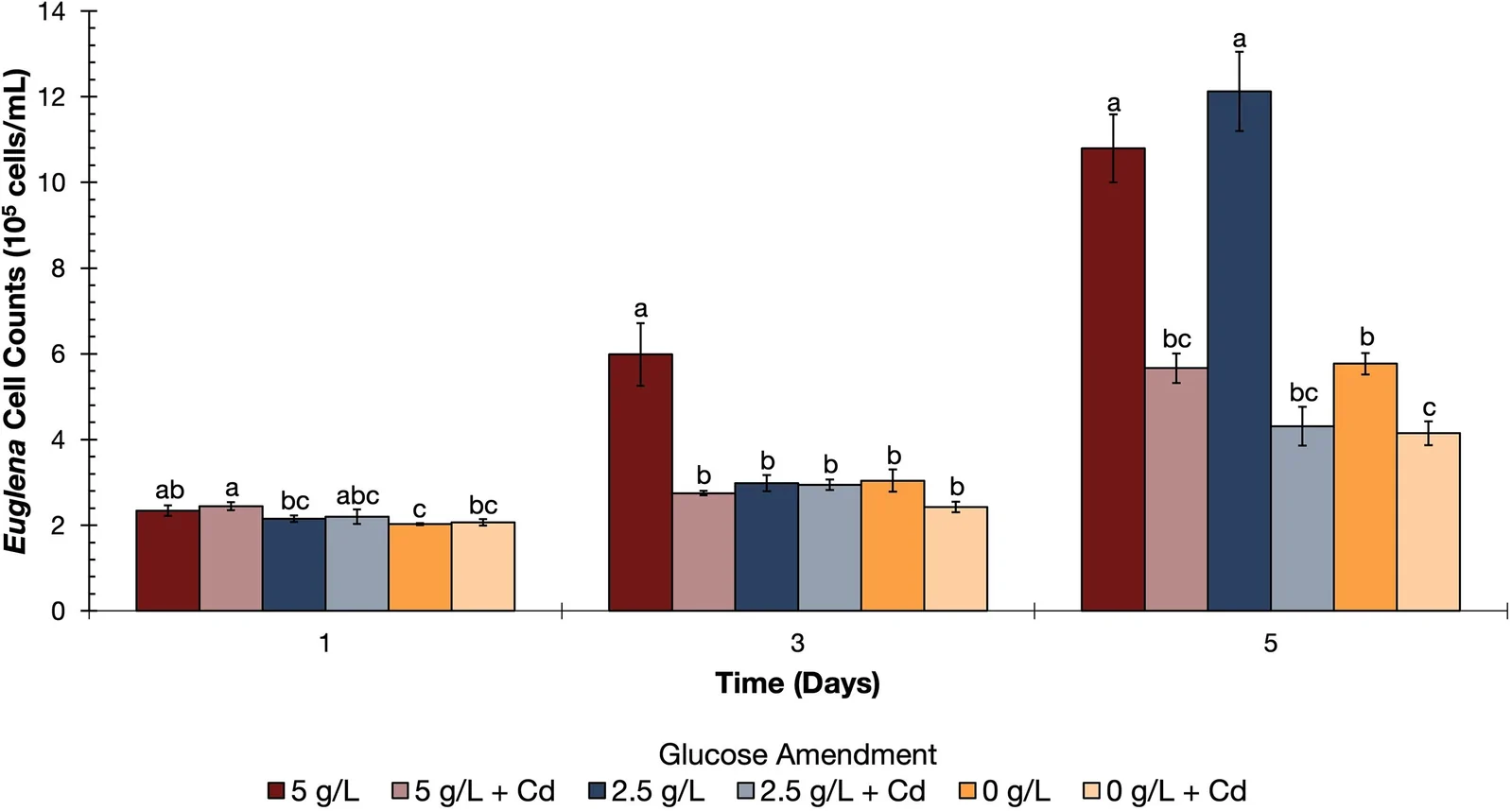
Glucose enhances the growth of *E. mutabilis*. Cultures of *E. mutabilis* were grown in for 5 days in MAM amended with glucose in the presence (light coloured bars) and absence (dark coloured bars) of CdCl_2_. Error bars represent standard deviation (*n* = 3). Statistical significance within each timepoint was assessed using a one-way ANOVA and Tukey’s HSD post-hoc test.

After 5 days, control cultures supplemented with glucose showed *E. mutabilis* cell counts approximately double those grown without glucose. Likewise—although not significant (*P* > 0.05)—cultures exposed to CdCl_2_ and supplemented with 5 g/l of glucose showed a 37% increase in cell counts compared to those exposed to CdCl_2_ without glucose, and a 31% increase compared to cultures grown with 2.5 g/l glucose and CdCl_2_.

After 5 days, chlorophyll content differed among glucose treatments under control conditions (Fig. S2), with both the 2.5 and 5 g/l glucose treatments exhibiting significantly lower chlorophyll content than the glucose-free control. In contrast, under CdCl_2_ exposure, chlorophyll content did not differ significantly between glucose-amended and glucose-free cultures. Thus, the glucose-associated reduction in chlorophyll observed under control conditions was not evident under CdCl_2_ stress.

Under control conditions, the combination of increased *E. mutabilis* cell abundance in the FAB (Fig. 2) and reduced chlorophyll content in glucose-amended cultures suggests that cell proliferation and chlorophyll accumulation were not directly coupled. This pattern is consistent with observations in other microalgae under glucose-supported mixotrophic conditions: increased growth can occur alongside reduced photosynthetic pigment content, potentially reflecting reduced reliance on carbon acquisition when organic carbon is readily available (Lang et al. 2017). However, because glucose also altered consortium structure and visible fungal proliferation, these responses should be interpreted as reflecting the integrated response of the FAB consortium rather than *E. mutabilis* metabolism alone.

As a metabolically flexible protist, *Euglena* can shift between phototrophic and heterotrophic metabolism (Ogbonna and Tanaka 2000). In the present study, glucose supplementation increased *E. mutabilis* cell counts in the FAB, particularly under CdCl_2_ stress, and also reduced the number of dead cells relative to cultures grown without glucose (Fig. S3), indicating that glucose contributed to growth and resilience within the FAB consortium (Marchello et al. 2018). Under CdCl_2_ stress, where photosynthetic efficiency may be impaired, glucose likely supported consortium metabolism through heterotrophic pathways, thereby sustaining cell proliferation despite metal toxicity.

The divergence between chlorophyll and cell abundance further indicates that growth and chlorophyll synthesis were not directly coupled in glucose-supplemented cultures. Under control conditions, glucose promoted overall growth after 5 days (Fig. 2), while several glucose treatments exhibited significantly reduced chlorophyll content relative to the glucose-free control (Fig. S2). This pattern is consistent with observations in other microalgae under glucose-supported mixotrophic conditions, where increased growth can occur alongside reduced photosynthetic pigment content, potentially reflecting reduced reliance on photosynthetic carbon acquisition when organic carbon is readily available (Lang et al. 2017). Under CdCl_2_ exposure, the relationship between glucose supplementation and chlorophyll content differed from that observed under control conditions. Glucose-amended cultures did not differ significantly in chlorophyll content from the glucose-free CdCl2 treatment at day 5, and chlorophyll content in the 2.5 g/l glucose + CdCl_2_ treatment was significantly higher than in the corresponding 2.5 g/l glucose treatment without CdCl_2_. This indicates that the reduction in chlorophyll associated with glucose supplementation under control conditions was not maintained during CdCl_2_ exposure. Because chlorophyll content alone does not measure photosynthetic activity, the physiological basis of this response cannot be resolved from the present data. Collectively, these results demonstrate that glucose supplementation altered the relationship between *E. mutabilis* abundance and chlorophyll content within the FAB consortium, with responses differing between control and CdCl_2_-exposed conditions. Increased *E. mutabilis* abundance under glucose supplementation was therefore not consistently associated with increased chlorophyll content, suggesting that consortium growth and chlorophyll accumulation responded differently to carbon availability and stress.

### Cellular CdCl_2_ accumulation varies with glucose supplementation

Total CdCl_2_ association with the harvested consortium biomass was quantified by ICP-MS and normalized to *E. mutabilis* cell abundance to facilitate comparisons among treatments (Fig. S4). This metric represents total consortium-associated CdCl_2_ rather than CdCl_2_ localized specifically within *E. mutabilis*. Negative controls grown without CdCl_2_ showed negligible CdCl_2_ signal by ICP-MS, confirming CdCl_2_ detected in CdCl_2_-exposed samples reflected treatment-derived accumulation rather than background contamination. Cellular CdCl_2_ accumulation in CdCl_2_-exposed samples increased markedly between days 1 and 3 in all treatments and remained elevated through day 5. Although the glucose-free treatment showed a numerically higher mean value at day 5, differences among glucose treatments were not statistically significant. Although glucose supplementation promoted microbial proliferation and flocculation elsewhere in the consortium, CdCl_2_ accumulation per 10^5^  *Euglena* cells did not differ significantly among glucose treatments. These data therefore do not support the conclusion that glucose increased CdCl_2_ accumulation efficiency, nor do they allow attribution of CdCl_2_ partitioning to *Talaromyces* or *A. acidophilum* under the conditions tested.

Previous studies indicated that intracellular distribution and physiological effects of CdCl_2_ in *Euglena* can vary with metabolic state. In *E. gracilis*, CdCl_2_ preferentially accumulates in chloroplasts under photoautotrophic conditions, whereas dark-grown cells accumulate CdCl_2_ primarily in mitochondria (Mendoza-Cozatl et al. 2002, Avilés et al. 2003). In addition, more than 60% of intracellular CdCl_2_ may be sequestered in chloroplasts, followed by redistribution associated with declines in chlorophyll content (Mendoza-Cozatl et al. 2002, Rodríguez-Zavala et al. 2007, Khatiwada et al. 2020). These findings suggest that trophic state may influence the intracellular handling and consequences of CdCl_2_ exposure; however, because no significant differences in total cellular CdCl_2_ accumulation were observed among glucose treatments in the present study, glucose is more likely to have affected stress tolerance, growth, and chlorophyll dynamics than CdCl_2_ uptake per se.

Beyond *E. mutabilis*, other members of the FAB consortium also contribute to heavy metal responses. *Talaromyces* contributes not only through surface adsorption of heavy metals (Romero et al. 2006, Nam et al. 2019, Guerra Sierra et al. 2022, Lu et al. 2024), but also by engaging in interspecies signalling that can trigger protective responses in co-cultured organisms under stress (Xiao et al. 2023). For example, *Talaromyces* strain MR1 enhanced CdCl_2_ accumulation in plants under high CdCl_2_ conditions while alleviating toxicity through the production of antioxidants and activation of host defence mechanisms (Xiao et al. 2023). In the present system, similar interactions may have contributed to consortium-level stress mitigation under CdCl_2_ exposure, although the current data do not demonstrate a significant glucose-dependent effect on cellular CdCl_2_ accumulation. Future work should quantify CdCl_2_ partitioning on a per-culture-volume basis together with independent estimates of fungal and bacterial biomass to determine how each consortium member contributes to metal retention within the FAB.

### Electron microscopy reveals close physical associations and unresolved intravacuole bodies within the FAB consortium

Scanning electron microscopy (SEM) provided visual evidence that the physical organization of the *E. mutabilis* FAB was strongly influenced by glucose availability. In cultures supplemented with 5 g/l (Fig. 3a and d) and 2.5 g/l (Fig. 3b and e) glucose, dense extracellular growth of rod-shaped bacterial cells and extensive fungal hyphae were observed, consistent with the proliferation of *A. acidophilum* and *Talaromyces*, respectively. Under these conditions, *E. mutabilis* cells appeared embedded within the complex extracellular microbial matrix. In contrast, cultures grown without glucose (Fig. 3c and f) lacked comparable visible extracellular bacterial or fungal structures. These observations indicate that glucose promoted extensive visible extracellular proliferation and physical association of the microbial partners within the FAB. Because SEM provides only a surface view of the consortium, the absence of visible microbial structures does not demonstrate that bacterial or fungal partners were absent, but rather that they were not detected as prominent extracellular populations under these conditions.

**Figure 3:**
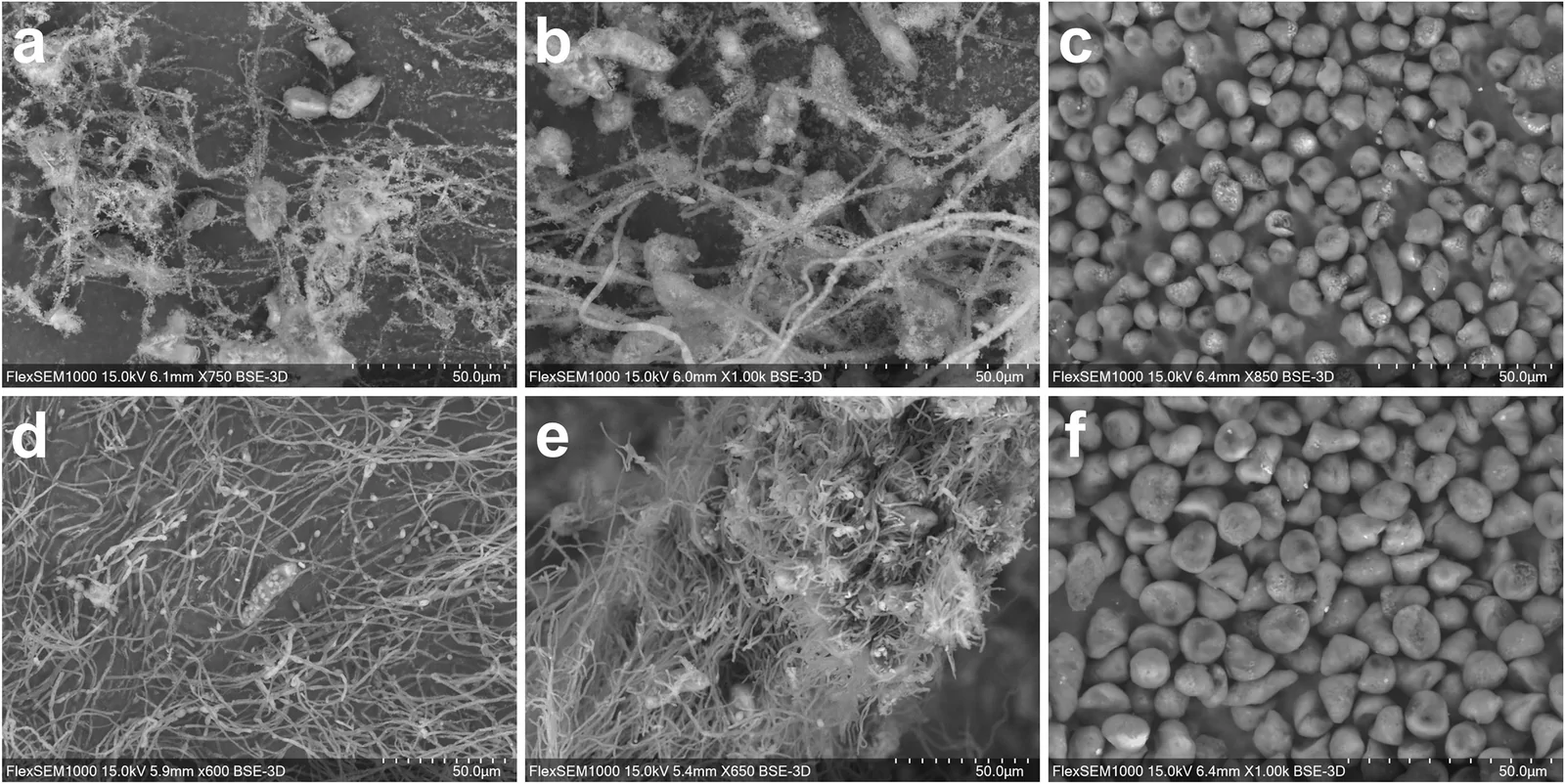
SEM images of the FAB consortium grown under carrying glucose concentrations with and without CdCl_2_. Cultures supplemented with 5 g/l (a) and 2.5 g/l (b) glucose showed abundant extracellular fungal hyphae and rod-shaped bacterial cells, whereas comparable structures were not observed in glucose-free cultures (c). Similar observations were noted in cultures supplemented 5 g/l + CdCl_2_ (d) and 2.5 g/l + CdCl_2_ (e) compared to CdCl_2_ only (f). SEM demonstrates treatment-dependent changes in consortium organization but does not determine the abundance or viability of individual consortium members.

Although SEM revealed treatment-dependent changes in consortium organization, fungal and bacterial abundance were not independently quantified. Future work combining microscopy with organism-specific molecular approaches would better resolve the contributions of each consortium member.

An additional feature of this consortium is its long-term stability under laboratory culture. Although *E. mutabilis* has been maintained in culture for several years, repeated attempts to establish axenic culture have not been successful, and the associated *Talaromyces* sp. and *Acidiphilium acidophilum* have remained persistent members of the consortium (Kaszecki et al. 2024). While these observations do not demonstrate obligate mutualism, they are consistent with a close ecological association among consortium members. The coordinated structural changes observed following glucose supplementation further support the hypothesis that interactions among consortium members contribute to community organization and stress adaptation. Determining whether this relationship represents facultative or obligate interdependence will require further studies in which individual consortium members can be selectively removed or manipulated.

Transmission electron microscopy (TEM) analysis of *E. mutabilis* cells revealed detailed ultrastructural features and the presence of intracellular, intravacuolar electron-dense bodies (Fig. 4). In cells cultured in MAM without CdCl_2_ exposure (Fig. 4a), characteristic organelles including the nucleus, chloroplasts, mitochondria, vacuoles, paramylon storage granules, and the pellicle were clearly visible. Similar structures were observed in cells grown in the presence of 100 µM CdCl_2_ (Fig. 4b), suggesting that basic cellular architecture is maintained under metal stress. In both treatments, multiple electron-dense bodies were consistently observed within vacuoles. These structures resemble ingested material or microbe-derived inclusions contained within food vacuoles (Akya et al. 2009, Buck et al. 2014), but they lacked definitive ultrastructural features, such as intact cell walls or recognizable prokaryotic organization, that would permit their identification. Consequently, TEM alone cannot determine whether these bodies represent intracellular microorganisms, degraded particulate material, or other cellular inclusions, and their biological significance remains unresolved. This interpretation is also consistent with the SEM observations, in which glucose-free cultures lacked clearly visible extracellular bacterial and fungal structures, although those data did not demonstrate that the cultures were axenic.

**Figure 4:**
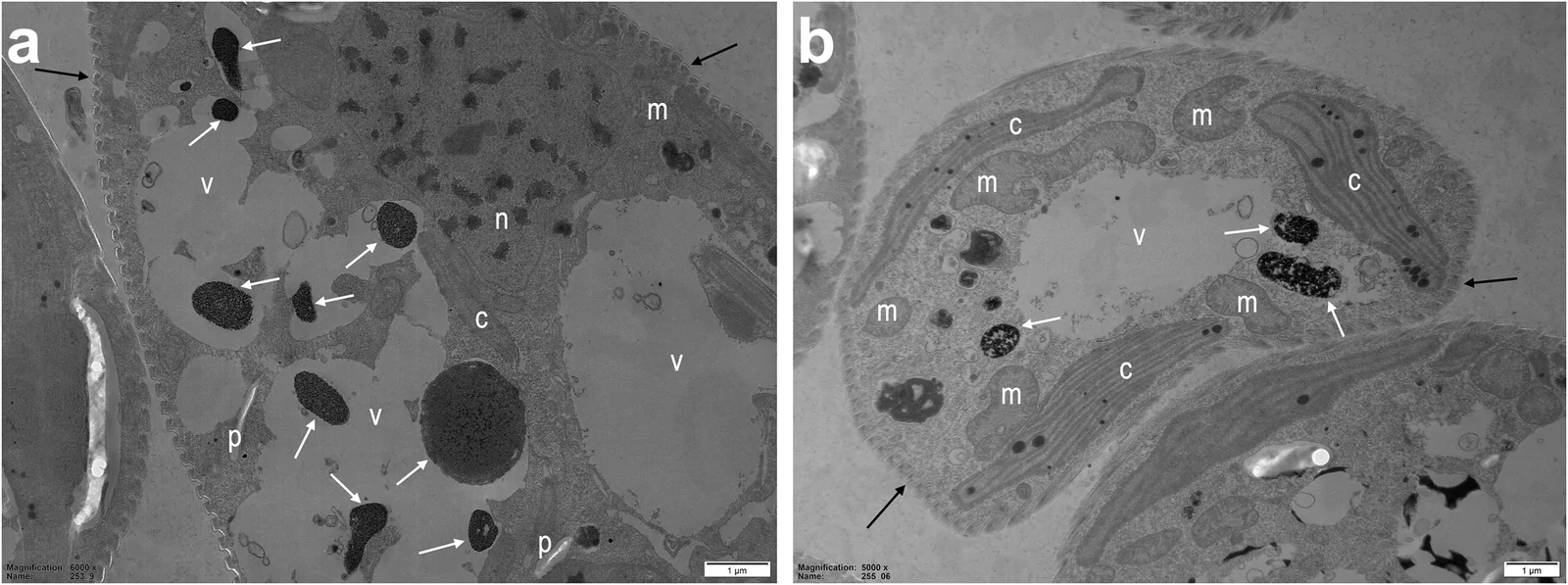
Transmission electron micrographs of *E. mutabilis* grown with and without CdCl_2_. Images of *E. mutabilis* cross-sections (scale bar represented 1 µm) grown in MAM (a) and MAM + 100 µM CdCl_2_ (b) readily display chloroplasts (c), mitochondria (m), the nucleus (n), paramylon (p), vacuoles (v), and the pellicle (black arrows). White arrows indicate electron-dense membrane-bound bodies within the vacuoles. These structures may represent ingested or degraded material within food vacuoles, but their identity cannot be resolved by TEM alone.

### Evaluation of FAB hormone production

The hormone profiles observed in this study represent the combined metabolic output of the *E. mutabilis*-associated fungal-algal-bacterial (FAB) consortium (Table 1). Since the culture cannot be maintained axenically without the loss of viability (Kaszecki et al. 2024), it is not possible to separate individual contributions to hormone production. Within this acidphilic assemblage, continuous metabolic exchange and signalling among consortium members likely shape the observed hormone pattern under heavy metal stress. Previous studies have demonstrated that *Euglena* spp., fungi, and bacteria each possess biosynthetic pathways for hormones, including salicylic acid (SA), cytokinins (CKs), and gibberellins (GAs) (Noble et al. 2014, Morrison et al. 2015, Kudoyarova et al. 2019, Nguyen et al. 2023). Therefore, the detection of these compounds in the FAB culture likely arises from overlapping biosynthetic processes and metabolic cross-talk among consortium members. While this study cannot definitively assign production to any specific organism, the hormone profiles can be interpreted as emergent properties of the consortium which reflect a coordinated strategy to maintain metabolic stability and community function under CdCl_2_ stress and varying glucose availability. Further studies using targeted isotopic labeling, single-organism isolation, or transcriptomic profiling would be required to confirm the specific source(s) of each hormone.

**Table 1:** Integrated summary of hormone dynamics in the FAB consortium under glucose and CdCl_2_ exposure.

Hormone	Main fraction	Condition of strongest accumulation	Temporal pattern	Putative source	Interpreted role in FAB
*t*Z	Supernatant	5 g/l glucose + CdCl_2_, day 5	Increases over time	Likely *Talaromyces*	Extracellular CK signalling under CdCl_2_ stress
*t*ZR	Supernatant	5 g/l glucose, day 5	Late peak	Precursor pool	Glucose-responsive precursor/interconversion state
*t*ZNT	Supernatant	0 g/l glucose, day 5	High at all time points	Likely *Talaromyces* precursor	Carbon-limited CdCl_2_ stress state
MeSZ	Supernatant	5 g/l glucose + CdCl_2_, day 5	Rises by day 3 then peaks	*Euglena* and/or consortium	Methyl-thiolated stress signalling
MeSZR	Supernatant	CdCl only, day 5	Late treatment-specific increase	*Euglena* and/or consortium	Alternate methyl-thiolated stress state
GA_7_	Supernatant >> pellet	Glucose + CdCl_2_	Strong increase over time	Likely *Talaromyces*	Extracellular GA signalling/stress adaptation
JA	Supernatant and pellet	Supernatant peak at 2.5 g/l glucose no CdCl_2_; pellet early in 5 g/l glucose	Transient	Likely *Euglena*	Stress adjustment
SA	Supernatant and pellet	Pellet high in glucose-free day 1; extracellular with glucose	Transient	Unresolved	Cooperative stress mitigation

For each hormone, the table indicates the principal fraction it was detected in, treatment condition associated with maximal accumulation, broad temporal trend, putative biological source within the consortium, and inferred physiological relevance in the context of glucose availability and CdCl_2_ stress. This summary is intended to emphasize the major cross-hormone patterns identified in this study, while detailed quantitative measurements and individual treatment comparisons are presented as supplementary figures.

#### Hormones associated with glucose and CdCl_2_ stress-adapted state

Hormone profiles in the *E. mutabilis* FAB varied markedly with glucose availability and CdCl_2_ exposure over time. The clearest hormone signature associated with the glucose-supported, Cd-exposed FAB state was the accumulation of extracellular bioactive CKs and GAs, together with increased methyl-thiolated CKs. Within the CK pool, *trans*-Zeatin (*t*Z) increased strongly over time in the culture supernatants of glucose-amended cultures, reaching its highest levels in the 5 g/l glucose + CdCl_2_ treatment by day 5, whereas only trace levels were detected in glucose-free cultures (Tables S5, S6; Fig. S5). Both *Euglena* and fungi can produce *c*Z through the tRNA-degradation pathways and *t*Z through the cytosolic mevalonate pathway (Hinsch et al. 2016, Bean et al. 2021, Anand et al. 2022), but *Euglena* reportedly produces only minute quantities of *t*Z (Noble et al. 2014). This suggests that *Talaromyces* is the more likely source of this hormone in the FAB consortium. The strong enrichment of *t*Z under glucose + CdCl_2_ was especially notable, consistent with previous work showing that exogenous *t*Z enhances heavy metal tolerance and uptake efficiency in *E. gracilis* under metal stress (Nguyen et al. 2023). Since *t*Z was not detected in the pellet, its accumulation in the supernatant may reflect extracellular release and intercellular signalling within the consortium (Gu et al. 2023).

A related pattern was observed for methylthiol-Zeatin (MeSZ), which rose substantially by day 3 in glucose-supplemented cultures and reached its highest level in the 5 g/l glucose + CdCl_2_ treatment by day 5 (Tables S7, S8; Fig. S6). Methyl-thiolated CKs are evolutionarily conserved compounds associated with stress responses, cellular communication, and microbe-host interactions (Kambhampati et al. 2017, Jorge et al. 2019, Prerostova et al. 2023). Since methyl-thiolated CKs have not generally been detected in axenic fungal cultures but have been reported in fungi associated with other microorganisms (Hinsch et al. 2015, Morrison et al. 2015), and because *Euglena* is known to produce methyl-thiolated CKs (Noble et al. 2014, Kuhne et al. 2023), the extracellular enrichment of MeSZ under glucose + CdCl_2_ is consistent with consortium-level signalling under stress. Its absence from the pellet further supports the idea of rapid extracellular accumulation.

GA_7_ followed the same general pattern and further supports the interpretation that the glucose + CdCl_2_ state is characterized by a fungal-associated extracellular hormone program. Of the GAs detected, GA_7_ showed the clearest response, with high levels in the supernatant of glucose-supplemented, Cd-exposed cultures and minimal accumulation in treatments lacking glucose or CdCl_2_ (Tables S9, S10; Fig. S7). Only a limited number of GAs are biologically active, and GA7 is recognized as the hormone associated with growth promotion and stress tolerance (Leitão and Enguita 2016). Endogenous GA production in *Euglena* remains largely uncharacterized (Bralczyk et al. 1978, Noble et al. 2014), whereas GA biosynthesis is widely attributed to fungi in co-culture systems (Khan and Lee 2013, El-Shahir et al. 2021). *Talaromyces* has been reported to produce gibberellins (Li et al. 2023), and related *Penicillium* species are well-known for synthesizing bioactive GAs (Khan and Lee 2013, Leitão and Enguita 2016, Salazar-Cerezo et al. 2018). In addition, GA_7_ has been identified as a dominant fungal GA (Fernández-Martn et al. 1995, Ahmad et al. 2010), and its biosynthetic pathway has so far only been attributed to fungi (Salazar-Cerezo et al. 2018). The co-occurence of GA_4_ with GA_7_ in Cd-treated supernatants further supports a fungal origin (Tables S9, S10; Fig. S7), as GA_4_ is the immediate precursor to GA_7_ in fungi (Salazar-Cerezo et al. 2018). Together, the coordinated enrichment of *t*Z, MeSZ, and GA_7_ under glucose + CdCl_2_ coincides with the increased visual fungal proliferation, enhanced flocculation, and improved persistence of *E. mutabilis* observed in glucose-supplemented cultures. Although these observations do not demonstrated that individual hormones directly mediate these phenotypes, they identify extracellular hormone accumulation as a biochemical feature consistently associated with the stress-adapted consortium state.

Jasmonic acid (JA) and SA also contributed to this integrated extracellular hormone profile (Tables S11, S12; Fig. S8). In plants, JA plays a central role in growth, development, and abiotic stress responses, including heavy metal stress (Ruan et al. 2019, Wang et al. 2020). Because *Euglena* is known to biosynthesize JA (Ueda et al. 1991), whereas fungal JA production is more commonly associated with pathogenic interactions (Eng et al. 2021), *E. mutabilis* is the most likely source of JA in the FAB. Although *Euglena* does not synthesize SA, it can perceive SA-related signals and respond through paramylon-associated effects (Scartazza et al. 2017).

#### Hormones showing contrasting or treatment-specific patterns

Although glucose + CdCl_2_ produced the clearest integrated extracellular hormone signature, not all compounds followed this pattern. The strongest exception was *trans*-Zeatin nucleotide (*t*ZNT), which accumulated primarily in CdCl_2_-treated cultures lacking glucose (Tables S5, S6; Fig. S5), reaching levels 10- to 100-fold greater than in other treatments. Because *t*ZNT is a precursor rather than a bioactive freebase, this pattern suggests that carbon limitation favours precursor accumulation rather than conversion to active CKs (Tudzynski et al. 2003).

A similar contrast was observed between MeSZ and MeSZR. Whereas MeSZ accumulated primarily in glucose-supplemented cultures, MeSZR accumulated mainly in the Cd-only treatment at day 5 (Tables S7, S8; Fig. S6). This divergence indicates that glucose availability influences CK metabolism in addition to overall hormone abundance (Kambhampati et al. 2017, Jorge et al. 2019, Prerostova et al. 2023).

Unlike the extracellular accumulation observed under glucose supplemenation, JA and SA displayed predominantly treatment-specific intracellular patterns (Tables S11, S12; Fig. S8). JA is most likely derived from *E. mutabilis* (Ueda et al. 1991), whereas the source of SA remains unresolved because fungal and bacterial partners may also contribute (Kilaru et al. 2007, Bari and Jones 2009, Chanclud and Morel 2016, Mishra and Baek 2021). In contrast to the extracellular CK and GA responses that accompanied increased visual fungal proliferation and flocculation, these intracellular JA and SA profiles were not consistently associated with visible changes in consortium structure. Instead, they were most consistent with cell-retained stress responses under carbon limitation rather than with the coordinated extracellular signalling observed when glucose is available.

Although the source of the individual hormones cannot be assigned to specific consortium members from the present data, the integrated extracellular hormone profiles provide a biochemical signature of consortium-level responses to glucose supplementation and CdCl_2_ stress. Rather than attributing hormone production to individual organisms, this study demonstrates that changes in community organization are accompanied by coordinated shifts in extracellular hormone composition. The coordinated extracellular enrichment of *t*Z, MeSZ, and GA_7_ under glucose + CdCl_2_ identifies these hormones and their associated biosynthetic pathways as priorities for future investigation. Organism-specific approaches will be required to determine their cellular sources and establish whether their accumulation contributes functionally to consortium responses under CdCl_2_ stress. From a bioremediation perspective, understanding how community-level hormone dynamics respond to carbon availability may ultimately assist in optimizing microbial consortia for improved persistence and performance in metal-contaminated environments.

#### Associations between hormone profiles and consortium phenotype

Collectively, the physiological, microscopy, and hormone data revealed associations among glucose availability, consortium organization, and extracellular hormone profiles during CdCl_2_ exposure. Glucose supplementation was associated with increased visible fungal and bacterial proliferation, enhanced flocculation, greater *E. mutabilis* abundance, and with altered extracellular accumulation of multiple hormones. These observations indicate that structural and biochemical responses occurred concurrently under increased carbon availability; however, the present data do not establish causal relationships among these responses. Because fungal and bacterial abundance was not independently quantified, the relative contribution of each consortium member cannot be resolved.

The observed hormone patterns can nevertheless be considered in the context of previous studies showing that CKs can enhance metal tolerance in *Euglena* (Nguyen et al. 2023), methyl-thiolated CKs are associated with stress adaptation and microbial interactions (Kambhampati et al. 2017, Jorge et al. 2019, Prerostova et al. 2023), and fungal-derived GAs contribute to growth regulation and stress responses (Khan and Lee 2013). In the present study, the extracellular *t*Z, MeSZ, and GA_7_ coincided with increased visible fungal proliferation and flocculation under glucose supplementation and CdCl_2_ exposure. These associations identify specific hormones for future targeted investigation but do not establish their cellular source or demonstrate that they directly contribute to the observed phenotype.

Glucose supplementation also altered consortium structure and hormone profiles without significantly increasing total consortium-associated CdCl_2_ per *E. mutabilis* cell. Thus, the observed extracellular hormone shifts appear to be associated with changes in consortium organization and physiological responses rather than increased CdCl_2_ accumulation. Further studies incorporating organism-specific quantitation, targeted manipulation of hormone pathways, and functional measurements will be required to determine whether these hormone shifts contribute directly to consortium responses to CdCl_2_ stress.

## Conclusions

Microscopy revealed changes in microbial organization under glucose availability and CdCl_2_ exposure, while TEM additionally revealed intravacuolar electron-dense bodies within *E. mutabilis*. The identity and biological significance of these bodies remain unresolved. Thes structural changes suggest that the FAB consortium undergoes reorganization in response to CdCl_2_ stress and glucose availability. Complementing these observations, hormone profiling showed that glucose and CdCl_2_ altered the extracellular accumulation of multiple hormones, including *t*Z, GA_7_, and methyl-thiolated CKs. Because these compounds have previously been implicated in microbial communication and stress responses, their coordinated accumulation is consistent with altered biochemical interactions within the consortium, although interspecies signalling was not directly demonstrated. Together, the microscopy and hormone data are consistent with the FAB functioning as a dynamic, adaptive community capable of coordinated structural and biochemical responses to environmental stress. Future work should aim to resolve the genetic basis of hormone biosynthesis in each FAB member, determine the source of individual hormones, and establish whether these compounds function directly in interspecies signalling or represent parallel physiological responses. Understanding these interactions provides a foundation for future studies investigating the use of natural FAB consortia in sustainable bioremediation and environmental resilience.

## Supplementary Material

xtag051_Supplemental_Files

## Data Availability

Data generated or analyzed during this study are provided in full within the published article and associated supplementary documents.
