# Supplementary material for: Glucose amendments are associated with structural and hormonal shifts in a fungal-algal-bacterial (FAB) consortium
Source: FEMS Microbes. 2026 Oct 7;7:xtag051. doi: 10.1093/femsmc/xtag051 (PMC13685799; doi:10.1093/femsmc/xtag051)
Supplement: Supplementary file 1 [file xtag051_supplemental_files.zip › EKaszecki_SupplementaryFile_Accepted.docx]

Table S1: Preliminary glucose screening used to select experimental treatment concentrations. Total chlorophyll content of the fungal-algal-bacterial (FAB) consortium culture in MAM medium supplemented with 0, 0.625, 1.25, 2.5, 5, 10, or 20 g/L glucose over 120 hours. Values are presented as mean ± SD (*n* = 3 independent biological replicates). Glucose treatments were analysed independently at each sampling time using one-way ANOVA followed by Tukey’s HSD test. Within each sampling time, treatments sharing one or more common letters are not significantly different (*p* > 0.05). The preliminary screening demonstrated that glucose supplementation produced concentration- and time-dependent changes in chlorophyll content, with both 2.5 and 5 g/L differing significantly from the glucose-free control. Concentrations above 5 g/L also produced extensive flocculation that interfered with reproducible sampling and quantitative enumeration of *E. mutabilis* and were therefore no selected for subsequent analysis.

|  | **Time** | | |
| --- | --- | --- | --- |
| **Glucose Concentration (g/L)** | **24 hours** | **72 hours** | **120 hours** |
| 0 | 0.671 ± 0.079 **a** | 1.068 ± 0.105 **a** | 1.456 ± 0.077 **ab** |
| 0.625 | 0.601 ± 0.036 **a** | 0.504 ± 0.061 **b** | 0.409 ± 0.035 **c** |
| 1.25 | 0.636 ± 0.046 **a** | 0.404 ± 0.029 **b** | 1.123 ± 0.095 **d** |
| 2.5 | 0.624 ± 0.049 **a** | 0.687 ± 0.099 **c** | 0.652 ± 0.050 **e** |
| 5 | 0.572 ± 0.036 **ab** | 0.744 ± 0.035 **c** | 1.345 ± 0.043 **af** |
| 10 | 0.565 ± 0.024 **ab** | 0.712 ± 0.048 **c** | 1.236 ± 0.074 **df** |
| 20 | 0.462 ± 0.031 **b** | 0.496 ± 0.014 **b** | 1.566 ± 0.111 **b** |
| Glucose concentration significantly affected total chlorophyll at 24 hours (F_6,14_ = 6.42, *p* = 0.0020), 72 hours (F_6,14_ = 35.22, *p* < 0.0001), and 120 hours (F_6,14_ = 93.05, *p* < 0.0001) | | | |

Figure S1: Calibration curve of cadmium (Cd^2+^) quantification using an Agilent 8900 ICP-QQQ-MS. Calibration was performed using a series of Cd^2+^ standards ranging from 1-500 ppb. The response (counts per second; CPS) showed a strong linear relationship with concentration (R^2^ = 0.9999), indicating strong instrumental performance and quantification accuracy. The regression equation (*y* = 8468.0768*x* + 0.0000E + 000) was used to determine Cd^2+^ concentrations in experimental samples. Detection limit (DL) and background equivalent concentration (BEC) were both 0 ppb, with a relative standard error (%RSE) of 4.3%.

Table S2: Hormones, listed in order of retention time (minutes) and group by included in the UHPLC-MS/MS method using PRM mode. 23 CKs (freebase, riboside, glucoside, methylated, and aromatic forms) and 8 acidic hormones were analyzed directly while 4 CK nucleotides were analyzed in their dephosphorylated riboside state.

| **Group** | **Analyte** | **Abbreviation** | **Labelled Analyte** |
| --- | --- | --- | --- |
| Freebases | *trans*-Zeatin | *t*Z | *t*Z-d_5_ |
|  | *cis*-Zeatin | *c*Z |  |
|  | Dihydrozeatin | DZ | DZ-d_3_ |
|  | Isopentyladenine | iP | iP-d_6_ |
| Ribosides | *trans*-Zeatin riboside | *t*ZR | *t*ZR-d_5_ |
|  | *cis*-Zeatin riboside | *c*ZR |  |
|  | Dihydrozeatin riboside | DZR | DZR-d_3_ |
|  | Isopentyladenosine | iPR | iPR-d_6_ |
| Nucleotides | *trans*‐zeatin riboside‐5′‐monophosphate | *t*ZNT | *t*ZR-d_5_ |
|  | *cis*‐zeatin riboside‐5′‐monophosphate | *c*ZNT |  |
|  | Dihydrozeatin riboside ‐5′‐monophosphate | DZNT | DZR-d_3_ |
|  | Isopentyladenosine‐5′monophosphate | iPNT | iPR-d_6_ |
| Methylthiols | 2‐Methylthio‐*trans*‐zeatin | MeSZ | MeSZ-d_5_ |
|  | 2‐Methylthio‐*trans*‐zeatin riboside | MeSZR | MeSZR-d_5_ |
|  | 2‐Methylthio‐isopentyladenine | MeSiP | MeSiP-d_6_ |
|  | 2‐Methylthio‐isopentyladenosine | MeSiPR | MeSiPR-d_6_ |
| Glucosides | *trans*‐zeatin‐O‐glucoside | *t*ZOG | *t*ZOG-d_5_ |
|  | *cis*‐zeatin‐O‐glucoside | *c*ZOG |  |
|  | Dihydrozeatin‐O‐glucoside riboside | DZOG | DZOG-d_7_ |
|  | *trans*‐zeatin‐7‐glucoside | *t*Z7G | *t*Z7G-d_5_ |
|  | *cis*‐zeatin‐7‐glucoside |  |  |
|  | *trans*‐zeatin‐9‐glucoside | *t*Z9G | *t*Z9G-d_5_ |
|  | *cis*‐zeatin‐9‐glucoside | *c*Z9G |  |
|  | Dihydrozeatin‐9‐glucoside | DZ9G | DZ9G-d_3_ |
|  | Isopentenyladenine‐7‐glucoside | iP7G | iP7G-d_6_ |
|  | Isopentenyladenine‐9‐glucoside | iP9G | iP7G-d_6_ |
| Aromatic CKs | Benzylaminopurine | BA | BA-d_7_ |
|  | Benzylaminopurine riboside | BAR | BAR-d_7_ |
| Acids | Abscisic acid | ABA | ABA-d_6_ |
|  | Gibberellin 1 | GA1 | GA1-d_2_ |
|  | Gibberellin 3 | GA3 | GA3-d_2_ |
|  | Gibberellic acid 4 | GA4 | GA4-d_2_ |
|  | Gibberellin 7 | GA7 | GA7-d_2_ |
|  | Indole-3-acetic acid | IAA | IAA-d_5_ |
|  | Salicylic acid | SA | SA-d_6_ |
|  | Jasmonic acid | JA | ABA-d_6_ |

Method S1: Detailed description of the method to extract, purify, and quantify hormones produced by the fungal-algal-bacterial (FAB) consortium grown in the presence and absence of glucose (± 100 µM CdCl_2_) according to the method described by Kisiala *et al* [1].

*Solid phase extraction and purification of hormones*

To facilitate hormone quantification via the isotope dilution technique, internal standards (IS) of deuterated hormones were added to each sample in 2 mL of 50% ice-cold acetonitrile (ACN). These included 60.9 ng ABA ([^2^H_4_] ABA) (PBI, Saskatoon, Canada), 10 ng each of IAA and SA (OLChemim, Olomouc, Czech Republic), and 10 ng each of the gibberellins GA_1_, GA_4_, GA_7_, GA_9_, and GA_20_. Additionally, 10 ng of deuterated CK standards were included, covering aromatic, methyl-thiolated, and glucoside forms. The CKs analyzed included benzyladenine (BA), benzyladenine riboside (BAR), kinetin (KIN), *cis*-Zeatin (cZ) and its derivatives (*c*ZR, *c*Z9G, *c*ZNT, *c*ZOG, *c*ZROG), dihydroZeatin (DZ) and its derivatives (DZNT, DZOG, DZR, DZROG, DZ9G), isopentenyladenine (iP) and its derivatives (iPNT, iP9G, iPR), as well as methyl-thiol and *trans*-Zeatin compounds (2MeSiP, 2MeSiPR, 2MeSZ, 2MeSZR, *t*Z, *t*ZR, *t*Z9G, *t*ZNT, *t*ZOG, *t*ZROG). While JA was not included as an internal standard, [^2^H_4_] ABA was used for JA quantification [2].

Dried cell pellets were resuspended with ice-cold 50% ACN in 2 mL Eppendorf tubes and agitated with two zirconium beads (Comeau Technique Ltd.) at 25 Hz for 5 minutes (Retsch Mixer Mill MM 400) in a 4℃ cold room. Supernatant and media blanks were resuspended in ice-cold 50% ACN and vortexed to mix. All samples were centrifuged and divided equally, with 1 mL of ACN transferred to a fresh 2 mL Eppendorf tube for acidic hormone analysis and the remaining 1 mL transferred to another tube for CK analysis. Additionally, three method-blank samples were extracted in parallel to the samples.

Samples designated for acidic hormone analysis were purified through HLB cartridges (Canadian Life Sciences Cat. No. VO34445). First, the columns were preconditioned under vacuum with analytical grade MeOH, B-Pure water (18.2 MΩ·cm), and 50% aqueous (v/v) ACN. After conditioning, samples were centrifuged (10 minutes at maximum speed), and the supernatant was added to each cartridge and eluted into 5 mL tubes by gravity followed with a final wash of 2 mL of 30% aqueous (v/v) ACN. Tubes for acidic hormones and CKs were evaporated to dryness in a vacuum concentrator (Savant UVS400) at ambient temperature.

Once dried, acidic hormone samples were derivatized and CK samples were stored in at -20℃. To each sample for acidic hormones, 75 µL of 1-propanol, 20 µL of B-Pure water, 5 μL of 500 mM bromocholine (TCI America Cat. No. B0577) in 70% ACN, and 1 μL of triethylamine (Fisher Scientific Cat. No. O4884-100) were added. Samples were incubated in a water bath at 80℃ for 130 minutes and then chilled on ice for 1 hour before being evaporated to dryness in a vacuum concentrator at ambient temperature. Dry samples were stored at -20℃ ahead of analysis.

Samples designated for CK analysis were redissolved in 1 mL of 1M Formic Acid (HCOOH) in preparation for purification through MCX cartridges (Canadian Life Sciences Cat. No. VO54445). Under vacuum, the columns were preconditioned with MeOH and HCOOH. After conditioning, samples were centrifuged (10 minutes at maximum speed), and the supernatant was added to each cartridge and eluted by gravity. With the vacuum reconnected the column was then equilibrated with HCOOH and MeOH. 5 mL of 0.35M ammonium hydroxide (NH_4_OH) was eluted through the cartridge under vacuum and collected in a 5 mL tube. This fraction will be used to assess the CKs in their nucleotide (NT) form. Next, 5 mL of 0.35 M NH_4_OH in 60% MeOH (H_2_O:MeOH 40:60 v/v) was eluted through the cartridge under vacuum and collected in a 5 mL tube. This fraction was used to assess CKs in their freebase (FB) and riboside (RB) forms. All collected fractions were evaporated to dryness in a vacuum concentrator at ambient temperature.

The dried samples for the FB fraction were stored at -20℃ ahead of analysis and the NT fraction was dephosphorylated to enable detection as ribosides by the ESI-MS/MS. Dried NT samples were redissolved in 1 mL of 0.1M ethanolamine (Sigma Aldrich Cat. No. E9508-100) and 3 units (12 µL) of phosphatase enzyme mix (New England Biolabs Cat. No. M0525L). Samples were incubated overnight at 37℃ and then evaporated to dryness in a vacuum concentrator at ambient temperature.

Dried NT samples were redissolved in 1.5 mL of B-Pure water in preparation for purification with C18 cartridges (Canadian Life Science Cat. No. IS72006). The columns were first preconditioned with MeOH and B-Pure water under vacuum. Then, samples were centrifuged (10 minutes at maximum speed), and the supernatant was added to each cartridge and eluted by gravity before being washed with B-Pure water. Final NT samples were collected by washing the column with 1.25 mL of 80% MeOH into 2 mL Eppendorf tubes. Collected samples were then evaporated to dryness in a vacuum concentrator at ambient temperature and stored at -20℃.

All final hormone fractions were redissolved in 300 µL of 0.08% acetic acid (AcOH) in 5% ACN. Samples were quickly vortexed to mix and centrifuged (3,724 RCF for 10 minutes) to remove any solid particles before transferring 250 µL to glass inserts in mass spectrometry vials.

*Hormone analysis by ultra high-performance liquid chromatography-electrospray ionization tandem mass spectrometry (UHPLC-ESI-MS/MS)*

Purified samples were analyzed for hormone content and concentration using ultra high-performance liquid chromatography-electrospray ionization-tandem mass spectrometry (UHPLC-(ESI)-MS/MS; Thermo Q-Exactive quadrupole orbitrap coupled with Thermo Dionex Ultimate 3000 UHPLC) as previously described [1]. Chromatographic separation of hormones was performed using an HGP-3400RS dual pump and a WPS-3000 autosampler, equipped with a Kinetex C18 column (2.1 mm internal diameter × 50 mm length, 2.6 µm particle size; Phenomenex, Torrance, CA, USA). The column was operated at an approximate room temperature of 22°C. Samples were injected at a volume of 25 µL and all hormone fractions were eluted using solution A (ddH₂O with 0.08% CH₃COOH) and solution B (CH₃CN with 0.08% CH₃COOH) at a flow rate of 0.5 mL^-1^ using a multi-step gradient. The initial condition of 5% solution B was maintained for 0.5 minutes, followed by a linear increase to 45% B over 4.5 minutes, then further increased to 95% B over the next 6.5 minutes. This condition was held for 1 minute before returning to the starting condition for 2 minutes to allow for column re-equilibration for a total run time of 8.2 minutes.

Instrument control and data acquisition were managed using Chromeleon 6.8 Chromatography Data System software (ThermoScientific, Ottawa, ON, Canada).

*Quantification of hormone profiles*

Quantification of hormone peaks was assessed using Xcalibur 3.0.63 software (ThermoFisher Scientific). Peaks corresponding to metabolites were initially identified by their monoisotopic mass as protonated molecules [M+H]^+^ and by comparing their RT to those of the IS. Extracted ion chromatograms (XICs) were generated for each hormone using the accurate masses of the two most intense fragment ions along with the unfragmented precursor ion, applying a mass tolerance of 3 ppm. Quantification was based on the area under the peak of each XIC. Quantified peaks from supernatant samples were normalized against the number of *E. mutabilis* cells in each volume of media (10 mL) and quantified peaks from pellet samples were normalized against dried weight.

| Table S3: Quantified hormone concentrations detected in the culture media under varying growth conditions. Only hormones present in the extracellular medium are reported; compounds not detected in the media have been excluded. | | | | | | | |
| --- | --- | --- | --- | --- | --- | --- | --- |
| **nmol/mL** | **Glucose [g/L]** | **Control** | | | **Cadmium** | | |
|  |  | Day 1 | Day 3 | Day 5 | Day 1 | Day 3 | Day 5 |
| ABA | 5 | 5.61 | 4.417 | 2.673 | 3.19 | 3.241 | 4.057 |
|  | 2.5 | 2.894 | 1.840 | 0.760 | 1.721 | 1.823 | 1.411 |
|  | 0 | 7.485 | 0.848 | 2.150 | 5.841 | 1.509 | 4.030 |
| SA | 5 | 567,680.54 | 633,978.565 | 682,269.486 | 1,448,339.61 | 400,372.699 | 352,517.862 |
|  | 2.5 | 236,205.310 | 306,631.529 | 189,767.506 | 235,611.617 | 296,674.132 | 90,960.888 |
|  | 0 | 23,335.500 | 10,268.070 | 12,474.069 | 37,192.728 | 20,443.743 | 1,090.713 |
| JA | 5 | 10,685.54 | 1,324.212 | 3,991.319 | 4,546.81 | n.d. | n.d. |
|  | 2.5 | n.d. | n.d. | n.d. | n.d. | 680.388 | n.d. |
|  | 0 | n.d. | n.d. | n.d. | 15.561 | n.d. | 32.023 |
| GA_4_ | 5 | n.d. | 69.626 | n.d. | 59.00 | n.d. | n.d. |
|  | 2.5 | n.d. | n.d. | n.d. | n.d. | 52.759 | n.d. |
|  | 0 | n.d. | n.d. | n.d. | n.d. | n.d. | n.d. |
| GA_7_ | 5 | 160.42 | 0.889 | 1.693 | 224.24 | n.d. | 155.870 |
|  | 2.5 | 86.782 | 0.416 | 0.604 | 99.599 | n.d. | 100.323 |
|  | 0 | 36.732 | 7.470 | 10.434 | 40.054 | 0.646 | 43.150 |

Figure S2: Glucose availability and Cd exposure jointly influence chlorophyll content of *E. mutabilis* FAB consortium. Cultures of *E. mutabilis* were grown for 5-days in MAM amended with glucose in the presence (light coloured bars) and absence (dark coloured bars) of CdCl_2_. Error bars represent standard deviation (*n* = 3). Statistical significance within each timepoint was assessed using a one-way ANOVA and Tukey’s HSD post-hoc test.

| Table S4: Post-stress viability of *Euglena mutabilis* and *Talaromyces* following 5 days of culture under varying glucose and Cd treatments. Data represent colony forming units (CFUs) recovered after 7 days on PDA at 22℃ in darkness (*n* = 3). Results are presented as semi-quantitative indicators of cell recovery and viability rather than quantitative toxicity measures to complement cell count, chlorophyll, and Cd accumulation data to collectively illustrate the resilience of the FAB under Cd stress. Before plating, samples were grown in their respective media types for 5-days. | | | | | |
| --- | --- | --- | --- | --- | --- |
|  | *E. mutabilis* | |  | *Talaromyces* | |
|  | MAM only | 100 µM CdCl_2_ |  | MAM only | 100 µM CdCl_2_ |
| 5 g/L | **+ +** | **+ +** |  | **+ + +** | **+ + +** |
| 2.5 g/L | **+ + +** | **+ +** |  | **+ + +** | **+ +** |
| 0 g/L | **+ +** | **+ +** |  | **+** | **+** |
| "-" if no CFU; "+" if < 50 CFU; "++" if 50><250 CFU; "+++" if >250 CFU or if a complete lawn is present and CFU count is impossible. | | | | | |

Figure S3: Dead cell counts of *E. mutabilis* in a FAB consortium exposed to Cd decrease when cells are grown in glucose. Cultures of *E. mutabilis* were grown for 5-days in MAM amended with glucose in the presence (light coloured bars) or absence (dark coloured bars) of CdCl_2_. The number of dead cells in each culture were determined using Trypan Blue dye and counting on a Hemocytometer. Error bars represent standard deviation (*n* = 3). Statistical significance within each timepoint was assessed using a one-way ANOVA and Tukey’s HSD post-hoc test.

Figure S4: Cadmium accumulation associated with a *E. mutabilis* FAB consortium following CdCl_2_ exposure. Cadmium accumulation is expressed as ng CdCl_2_ per 10^5^ *Euglena* cells for cultures supplemented with 0, 2.5, or 5 g/L glucose. Faint points represent individual biological replicates (*n* = 3), whereas larger points and dashed lines indicate treatment means across time; error bars denote standard deviation. Cellular CdCl_2_ accumulation increased markedly between days 1 and 3 in all treatments and remained elevated through day 5. Although mean CdCl_2_ accumulation differed numerically among treatments at later timepoints, no statistically significant differences were detected among glucose treatments.

| Table S5: Quantified CK concentrations in the culture supernatant across experimental treatments. Values represent the mean concentration (± standard error, *n* = 3) of each sample measured at indicated time points. Treatments include varying glucose amendments with and without Cd exposure. Data reflect extracellular CK accumulation as part of the experimental assessment of environmental and nutrient stress on CK production. | | | | | | | | | |
| --- | --- | --- | --- | --- | --- | --- | --- | --- | --- |
| **fmol/mL** | **Glucose [g/L]** | **Control** | | | **Change** | **Cadmium** | | | **Change** |
|  |  | Day 1 | Day 3 | Day 5 |  | Day 1 | Day 3 | Day 5 |  |
| *t*Z | 5 | n.d. | 86.918 ±  5.299 | 40.311 ±  2.324 | ↑ | 6.997 ±  2.038 | 130.847 ±  18.076 | 234.789 ±  87.413 | ↑ |
|  | 2.5 | 2.957 ±  0.861 | 129.068 ±  11.914 | 21.493 ±  4.891 | ↑ | n.d. | 51.818 ±  9.097 | 145.986 ±  49.169 | ↑ |
|  | 0 | n.d. | n.d. | n.d. | − | n.d. | 3.247 ±  1.093 | n.d. | − |
| *t*ZR | 5 | 1.205 ±  0.363 | 2.263 ±  0.982 | 149.072 ±  20.789 | ↑ | 20.532 ±  1.695 | 7.265 ±  1.042 | n.d. | ↓ |
|  | 2.5 | n.d. | 3.572 ±  1.659 | 18.259 ±  5.287 | ↑ | 7.959 ±  2.079 | 12.588 ±  3.555 | n.d. | ↓ |
|  | 0 | n.d. | 3.054 ±  0.928 | 2.641 ±  1.077 | ↑ | 6.068 ±  1.556 | 16.661 ±  2.013 | n.d. | ↓ |
| *t*ZNT | 5 | n.d. | n.d. | 34.335 ±  10.844 | ↑ | 51.621 ±  24.409 | n.d. | 79.771 ±  23.045 | ↑ |
|  | 2.5 | n.d. | n.d. | 15.307 ±  1.283 | ↑ | n.d. | 47.610 ±  8.797 | 3,333.622 ±  1,335.907 | ↑ |
|  | 0 | n.d. | n.d. | n.d. | − | 133.177 ±  11.857 | n.d. | n.d. | ↓ |
| *c*Z | 5 | n.d. | 29.635 ±  14.548 | 207.019 ±  19.130 | ↑ | n.d. | n.d. | 120.666 ±  59.072 | ↑ |
|  | 2.5 | n.d. | 16.183 ±  8.448 | 131.264 ±  14.583 | ↑ | n.d. | 14.578 ±  3.856 | 265.705 ±  89.104 | ↑ |
|  | 0 | n.d. | n.d. | n.d. | − | n.d. | n.d. | n.d. | − |
| *c*ZNT | 5 | n.d. | n.d. | n.d. | − | 59.058 ±  23.486 | 95.326 ±  26.337 | 140.199 ±  18.734 | ↑ |
|  | 2.5 | n.d. | n.d. | 15.730 ±  4.828 | ↑ | 18.455 ±  10.655 | 64.321 ±  19.994 | 3,389.643 ±  947.008 | ↑ |
|  | 0 | n.d. | n.d. | n.d. | − | 77.239 ±  24.759 | 133.526 ±  53.396 | n.d. | ↓ |
| iP | 5 | n.d. | 3.433 ±  0.361 | 1.390 ±  0.430 | ↑ | n.d. | 2.444 ±  0.188 | n.d. | − |
|  | 2.5 | 1.446 ±  0.405 | 2.219 ±  0.850 | n.d. | ↓ | n.d. | 4.022 ±  0.776 | n.d. | − |
|  | 0 | n.d. | 1.018 ±  0.060 | n.d. | − | n.d. | n.d. | 2.614 ±  0.774 | ↑ |
| iPR | 5 | n.d. | 2.341 | n.d. | − | 3.332 ±  0.299 | 10.647 ±  3.467 | n.d. | ↓ |
|  | 2.5 | n.d. | 1.244 | n.d. | − | 2.561 ±  0.590 | 3.673 ±  0.491 | n.d. | ↓ |
|  | 0 | n.d. | 0.988 | n.d. | − | n.d. | 4.518 ±  0.446 | 30.642 ±  9.386 | ↑ |

| Table S6: Quantified CK concentrations in culture pellets across experimental treatments. Values represent the mean concentration (± standard error, *n* = 3) of each sample measured at indicated time points. Treatments include varying glucose amendments with and without Cd exposure. Data reflect intracellular CK accumulation as part of the experimental assessment of environmental and nutrient stress on CK production. | | | | | | | | | | |
| --- | --- | --- | --- | --- | --- | --- | --- | --- | --- | --- |
| **fmol/g [DW]** | **Glucose**  **[g/L]** | **Control** | | | **Change** | **Cadmium** | | | **Change** |  |
|  |  | Day 1 | Day 3 | Day 5 |  | Day 1 | Day 3 | Day 5 |  |  |
| *t*Z | 5 | n.d. | n.d. | n.d. | − | n.d. | n.d. | n.d. | − |  |
|  | 2.5 | n.d. | n.d. | n.d. | − | n.d. | n.d. | n.d. | − |  |
|  | 0 | n.d. | n.d. | n.d. | − | n.d. | n.d. | n.d. | − |  |
| *t*ZR | 5 | n.d. | n.d. | 349.657 ±  26.351 | ↑ | n.d. | n.d. | 520.286 ±  148.531 | ↑ |  |
|  | 2.5 | n.d. | n.d. | 268.235 ±  36.009 | ↑ | n.d. | n.d. | 600.748 ±  332.808 | ↑ |  |
|  | 0 | n.d. | n.d. | n.d. | − | n.d. | n.d. | n.d. | − |  |
| *t*ZNT | 5 | n.d. | n.d. | 21.398 ±  6.634 | ↑ | n.d. | n.d. | 17.911 ±  5.210 | ↑ |  |
|  | 2.5 | 1,972.490 ±  983.172 | 53.585 ±  28.570 | 113.321 ±  19.537 | ↓ | n.d. | n.d. | n.d. | − |  |
|  | 0 | n.d. | 243.179 ±  32.772 | 216.652 ±  64.474 | ↑ | n.d. | n.d. | n.d. | − |  |
| *c*Z | 5 | n.d. | n.d. | 13.863 ±  4.866 | ↑ | n.d. | n.d. | n.d. | − |  |
|  | 2.5 | n.d. | n.d. | 8.017 ±  3.454 | ↑ | n.d. | n.d. | n.d. | − |  |
|  | 0 | n.d. | n.d. | n.d. | − | n.d. | n.d. | n.d. | − |  |
| *c*ZNT | 5 | n.d. | 38.629 ±  22.302 | 25.280 ±  4.715 | ↑ | n.d. | 73.857 ±  38.402 | 200.603 ±  57.963 | ↑ |  |
|  | 2.5 | n.d. | 122.196 ±  70.550 | 39.293 ±  22.686 | ↑ | n.d. | 187.686 ±  69.105 | 346.821 ±  141.058 | ↑ |  |
|  | 0 | n.d. | 116.764 ±  67.414 | n.d. | − | n.d. | n.d. | 63.302 ±  3.187 | ↑ |  |
| iP | 5 | 39.135 ±  11.914 | 2.522 ±  0.746 | n.d. | ↓ | n.d. | n.d. | 13.557 ±  5.581 | ↑ |  |
|  | 2.5 | n.d. | 2.339 ±  0.729 | n.d. | − | n.d. | n.d. | n.d. | − |  |
|  | 0 | n.d. | n.d. | n.d. | − | n.d. | n.d. | n.d. | − |  |
| iPR | 5 | n.d. | 1.691 ±  0.505 | 100.107 ±  3.763 | ↑ | n.d. | n.d. | n.d. | − |  |
|  | 2.5 | n.d. | n.d. | 101.023 ±  17.140 | ↑ | n.d. | n.d. | 118.496 ±  52.838 | ↑ |  |
|  | 0 | n.d. | n.d. | n.d. | − | n.d. | n.d. | n.d. | − |  |

Figure S5: Glucose and CdCl_2_ exposure influence CK dynamics in the FAB consortium. Supernatant concentrations (fmol/mL) of *t*Z (a), *t*ZR (b), and *t*ZNT (c) from a *E. mutabilis* FAB grown in varying concentration of glucose in the presence and absence of CdCl_2_ over 5-days. Error bars represent standard deviation (*n* = 3). Statistical significance within each timepoint was assessed using a one-way ANOVA and Tukey’s HSD post-hoc test including cultures where the hormones were not detected.

| Table S7: Quantified methyl-thiolated CK concentrations in the culture supernatant across experimental treatments. Values represent the mean concentration (± standard error, *n* = 3) of each sample measured at indicated time points. Treatments include varying glucose amendments with and without Cd exposure. Data reflect extracellular methylated CK accumulation as part of the experimental assessment of environmental and nutrient stress on methylated CK production. | | | | | | | | | |
| --- | --- | --- | --- | --- | --- | --- | --- | --- | --- |
| **fmol/mL** | **Glucose [g/L]** | **Control** | | | **Change** | **Cadmium** | | | **Change** |
|  |  | Day 1 | Day 3 | Day 5 |  | Day 1 | Day 3 | Day 5 |  |
| MeSZ | 5 | 12.948 ±  0.633 | 137.823 ±  16.279 | 76.492 ±  2.731 | ↑ | n.d. | 185.381 ±  7.025 | 612.868 ±  257.268 | ↑ |
|  | 2.5 | 18.801 ±  1.288 | 187.489 ±  8.747 | 56.142 ±  3.605 | ↑ | n.d. | 75.358 ±  5.220 | n.d. | − |
|  | 0 | n.d. | 31.493 ±  1.561 | 32.708 ±  4.432 | ↑ | n.d. | n.d. | n.d. | − |
| MeSZR | 5 | 2.485 ±  0.416 | 9.399 ±  1.256 | n.d. | ↓ | 11.150 ±  1.003 | 18.204 ±  5.963 | 37.494 ±  8.841 | ↑ |
|  | 2.5 | 2.229 ±  0.319 | 19.083 ±  0.915 | n.d. | ↓ | 4.517 ±  0.742 | 10.195 ±  0.602 | n.d. | ↓ |
|  | 0 | 2.551 ±  0.186 | 13.919 ±  1.720 | 18.050 ±  0.184 | ↑ | 2.965 ±  0.326 | 14.793 ±  0.836 | n.d. | ↓ |

| Table S8: Quantified methyl-thiolated CK concentrations in culture pellets across experimental treatments. Values represent the mean concentration (± standard error, *n* = 3) of each sample measured at indicated time points. Treatments include varying glucose amendments with and without Cd exposure. Data reflect intracellular methylated CK accumulation as part of the experimental assessment of environmental and nutrient stress on methylated CK production. | | | | | | | | | | |
| --- | --- | --- | --- | --- | --- | --- | --- | --- | --- | --- |
| **fmol/g [DW]** | **Glucose [g/L]** | **Control** | | | **Change** | **Cadmium** | | | **Change** |  |
|  |  | Day 1 | Day 3 | Day 5 |  | Day 1 | Day 3 | Day 5 |  |  |
| MeSZ | 5 | n.d. | n.d. | n.d. | − | n.d. | n.d. | n.d. | − |  |
|  | 2.5 | n.d. | n.d. | n.d. | − | n.d. | n.d. | n.d. | − |  |
|  | 0 | n.d. | n.d. | n.d. | − | n.d. | n.d. | n.d. | − |  |
| MeSZR | 5 | n.d. | n.d. | n.d. | − | n.d. | n.d. | n.d. | − |  |
|  | 2.5 | n.d. | n.d. | n.d. | − | n.d. | n.d. | n.d. | − |  |
|  | 0 | n.d. | n.d. | n.d. | − | n.d. | n.d. | n.d. | − |  |

Figure S6: Glucose and CdCl_2_ affect methyl-thiolated Zeatin derivatives in FAB culture. Supernatant concentrations (pmol/mL) of MeSZ (a) and MeSZR (b) from a *E. mutabilis* FAB grown in varying concentration of glucose in the presence and absence of CdCl_2_ over 5-days. Error bars represent standard deviation (*n* = 3). Statistical significance within each timepoint was assessed using a one-way ANOVA and Tukey’s HSD post-hoc test including cultures where the hormones were not detected.

| Table S9: Quantified gibberellin concentrations in the culture supernatant across experimental treatments. Values represent the mean concentration (± standard error, *n* = 3) of each sample measured at indicated time points. Treatments include varying glucose amendments with and without Cd exposure. Data reflect extracellular gibberellin accumulation as part of the experimental assessment of environmental and nutrient stress on gibberellin production. | | | | | | | | | |
| --- | --- | --- | --- | --- | --- | --- | --- | --- | --- |
| **fmol/mL** | **Glucose [g/L]** | **Control** | | | **Change** | **Cadmium** | | | **Change** |
|  |  | Day 1 | Day 3 | Day 5 |  | Day 1 | Day 3 | Day 5 |  |
| GA_3_ | 5 | n.d. | n.d. | 8.770 ±  1.234 | ↑ | n.d. | n.d. | n.d. | − |
|  | 2.5 | n.d. | n.d. | 68.224 ±  38.779 | ↑ | n.d. | n.d. | n.d. | − |
|  | 0 | n.d. | n.d. | 2.143 ±  0.122 | ↑ | n.d. | n.d. | n.d. | − |
| GA_4_ | 5 | n.d. | n.d. | n.d. | − | n.d. | 180,399.436 ±  97,985.708 | 19,398.803 ±  7,891.018 | ↑ |
|  | 2.5 | n.d. | n.d. | n.d. | − | n.d. | 157,932.445 ±  50,227.927 | 20,797.342 ±  10,997.882 | ↓ |
|  | 0 | n.d. | n.d. | n.d. | − | n.d. | n.d. | n.d. | − |
| GA_7_ | 5 | n.d. | n.d. | n.d. | − | 1,544.157 ±  269.850 | 84.335 ±  9.537 | n.d. | ↓ |
|  | 2.5 | n.d. | 5.319 ±  3.071 | 31.574 ±  12.862 | ↑ | 3,402.130 ±  919.111 | 3,965.852 ±  2,264.589 | 23,409.480 ±  2,074.459 | ↑ |
|  | 0 | n.d. | n.d. | n.d. | − | n.d. | n.d. | n.d. | − |

| Table S10: Quantified gibberellin concentrations in culture pellets across experimental treatments. Values represent the mean concentration (± standard error, *n* = 3) of each sample measured at indicated time points. Treatments include varying glucose amendments with and without Cd exposure. Data reflect intracellular gibberellin accumulation as part of the experimental assessment of environmental and nutrient stress on gibberellin production. | | | | | | | | | |
| --- | --- | --- | --- | --- | --- | --- | --- | --- | --- |
| **fmol/g [DW]** | **Glucose [g/L]** | **Control** | | | **Change** | **Cadmium** | | | **Change** |
|  |  | Day 1 | Day 3 | Day 5 |  | Day 1 | Day 3 | Day 5 |  |
| GA_3_ | 5 | n.d. | n.d. | n.d. | − | n.d. | n.d. | n.d. | − |
|  | 2.5 | n.d. | n.d. | n.d. | − | n.d. | n.d. | n.d. | − |
|  | 0 | n.d. | n.d. | n.d. | − | n.d. | n.d. | n.d. | − |
| GA_4_ | 5 | n.d. | n.d. | n.d. | − | n.d. | n.d. | n.d. | − |
|  | 2.5 | n.d. | n.d. | n.d. | − | n.d. | n.d. | n.d. | − |
|  | 0 | n.d. | n.d. | n.d. | − | n.d. | n.d. | n.d. | − |
| GA_7_ | 5 | 10,690.680 ±  956.556 | 504.811 ±  261.805 | 45.982 ±  13.085 | ↓ | 22,236.410 ±  3,409.701 | 74.025 ±  23.717 | 39.449 ±  3.297 | ↓ |
|  | 2.5 | 9,631.789 ±  1,885.649 | 27.921 ±  0.462 | 129.966 ±  55.723 | ↓ | 18,737.977 ±  2,091.436 | 123.383 ±  37.687 | 85.619 ±  15.959 | ↓ |
|  | 0 | 3,493.200 ±  338.149 | 68.753 ±  5.739 | 51.051 ±  3.183 | ↓ | 17,769.584 ±  2,824.381 | 186.210 ±  49.764 | 131.189 ±  31.573 | ↓ |

Figure S7: Gibberellin (GA_7_) distribution between supernatant and pellet is influenced by glucose and CdCl_2_. Supernatant (a) and pellet (b) concentrations (fmol/mL and fmol/mg DW) of GA_7_ from a *E. mutabilis* FAB grown in varying concentration of glucose in the presence and absence of CdCl_2_ over 5-days. Error bars represent standard deviation (*n* = 3). Statistical significance within each timepoint was assessed using a one-way ANOVA and Tukey’s HSD post-hoc test including cultures where the hormones were not detected.

| Table S11: Quantified acidic hormone concentrations in the culture supernatant across experimental treatments. Values represent the mean concentration (± standard error, *n* = 3) of each sample measured at indicated time points. Treatments include varying glucose amendments with and without Cd exposure. Data reflect extracellular acidic hormone accumulation as part of the experimental assessment of environmental and nutrient stress on acidic hormone production. | | | | | | | | | |
| --- | --- | --- | --- | --- | --- | --- | --- | --- | --- |
| **pmol/mL** | **Glucose [g/L]** | **Control** | | | **Change** | **Cadmium** | | | **Change** |
|  |  | Day 1 | Day 3 | Day 5 |  | Day 1 | Day 3 | Day 5 |  |
| ABA | 5 | 1.076 ±  0.158 | 2.780 ±  0.406 | 0.952 ±  0.150 | ↓ | 0.171 ±  0.028 | 8.085 ±  4.350 | 2.857 ±  1.064 | ↑ |
|  | 2.5 | 0.833 ±  0.106 | 3.382 ±  0.232 | 0.402 ±  0.134 | ↓ | 0.286 ±  0.037 | 7.715 ±  2.066 | 17.487 ±  3.280 | ↑ |
|  | 0 | 0.163 ±  0.024 | 0.448 ±  0.053 | 0.320 ±  0.065 | ↑ | n.d. | 0.603 ±  0.066 | 0.745 ±  0.041 | ↑ |
| IAA | 5 | 3.470 ±  0.179 | 7.569 ±  0.399 | 0.753 ±  0.237 | ↓ | 5.261 ±  0.356 | 32.347 ±  1.298 | 17.531 ±  6.894 | ↑ |
|  | 2.5 | 3.828 ±  0.080 | 12.526 ±  1.416 | n.d. | ↓ | 5.184 ±  0.030 | 26.252 ±  3.182 | n.d. | ↓ |
|  | 0 | 2.194 ±  0.035 | 3.533 ±  0.316 | 1.081 ±  0.146 | ↓ | 6.563 ±  0.017 | 17.798 ±  2.324 | 8.347 ±  1.175 | ↑ |
| JA | 5 | n.d. | 122.025 ±  27.938 | n.d. | − | 11.922 ±  6.883 | 706.392 ±  129.929 | 32.973 ±  15.203 | ↑ |
|  | 2.5 | 189.563 ±  58.931 | 1,751.943 ±  819.498 | n.d. | ↓ | 50.636 ±  18.281 | 1,191.295 ±  687.794 | 866.985 ±  81.061 | ↑ |
|  | 0 | n.d. | n.d. | n.d. | − | n.d. | n.d. | n.d. | − |
| SA | 5 | 247,881.983 ±  110,415.971 | 28,015.481 ±  2,596.282 | 19,287.359 ±  11,135.562 | ↓ | 15,943.776 ±  6,352.508 | 236,085.369 ±  36,316.032 | 17,990.354 ±  2,048.180 | ↑ |
|  | 2.5 | 21,301.229 ±  5,571.372 | 248,546.777 ±  103,358.373 | n.d. | ↓ | 14,875.196 ±  787.820 | 780,674.129 ±  252,116.032 | 125,914.032 ±  5,696.654 | ↑ |
|  | 0 | 2,850.578 ±  1,001.906 | 372.308 ±  156.192 | 224.440 ±  57.320 | ↓ | 220.517 ±  72.220 | 297.049 ±  93.276 | 711.275 ±  66.177 | ↑ |

| Table S12: Quantified acidic hormone concentrations in culture pellets across experimental treatments. Values represent the mean concentration (± standard error, *n* = 3) of each sample measured at indicated time points. Treatments include varying glucose amendments with and without Cd exposure. Data reflect intracellular acidic hormone accumulation as part of the experimental assessment of environmental and nutrient stress on acidic hormone production. | | | | | | | | | |
| --- | --- | --- | --- | --- | --- | --- | --- | --- | --- |
| **pmol/g [DW]** | **Glucose [g/L]** | **Control** | | | **Change** | **Cadmium** | | | **Change** |
|  |  | Day 1 | Day 3 | Day 5 |  | Day 1 | Day 3 | Day 5 |  |
| ABA | 5 | 12.814 ±  1.756 | 2.028 ±  0.299 | 2.636 ±  0.351 | ↓ | 5.756 ±  1.324 | 0.367 ±  0.075 | 0.161 ±  0.016 | ↓ |
|  | 2.5 | 15.761 ±  4.713 | 0.955 ±  0.238 | 2.599 ±  0.014 | ↓ | 5.932 ±  1.578 | 0.344 ±  0.107 | 0.159 ±  0.035 | ↓ |
|  | 0 | 4.723 ±  0.355 | 7.231 ±  1.343 | 5.621 ±  1.238 | ↑ | 4.026 ±  0.288 | 1.400 ±  0.499 | 2.364 ±  0.716 | ↓ |
| IAA | 5 | 1.286 ±  0.049 | 0.791 ±  0.068 | 3.888 ±  0.193 | ↑ | n.d. | 1.444 ±  0.175 | 8.355 ±  0.942 | ↑ |
|  | 2.5 | 2.020 ±  0.646 | 0.880 ±  0.046 | 5.121 ±  0.490 | ↑ | n.d. | 1.078 ±  0.137 | 4.332 ±  0.817 | ↑ |
|  | 0 | 0.557 ±  0.098 | 1.114 ±  0.181 | 3.036 ±  0.497 | ↑ | n.d. | n.d. | 1.411 ±  0.191 | ↑ |
| JA | 5 | 29,986.958 ±  13,055.410 | 501.430 ±  9.730 | 107.563 ±  26.654 | ↓ | 5,230.309 ±  954.169 | 894.898 ±  202.984 | n.d. | ↓ |
|  | 2.5 | 10,976.027 ±  2,456.056 | 70.459 ±  25.996 | 123.150 ±  31.847 | ↓ | 2,285.386 ±  670.090 | 607.142 ±  141.289 | n.d. | ↓ |
|  | 0 | 2,787.800 ±  1,061.575 | 3,285.327 ±  1,303.349 | 390.771 ±  70.113 | ↓ | 3,910.259 ±  570.027 | 2,828.120 ±  456.248 | 1,168.303 ±  374.982 | ↓ |
| SA | 5 | 225,044.465 ±  58,072.318 | 31,830.248 ±  3,732.080 | 1,302.979 ±  341.306 | ↓ | 140,719.359 ±  21,216.086 | 47,857.399 ±  11,076.129 | 1,013.295 ±  73.395 | ↓ |
|  | 2.5 | 155,301.476 ±  22,528.209 | 8,305.608 ±  1,467.990 | 4,908.685 ±  1,403.061 | ↓ | 59,578.801 ±  17,199.387 | 46,723.513 ±  10,638.970 | 524.686 ±  411.758 | ↓ |
|  | 0 | 45,145.799 ±  7,872.895 | 125,446.832 ±  31,841.829 | 24,698.259 ±  2,425.237 | ↓ | 101,762.493 ±  11,108.919 | 82,524.811 ±  20,749.825 | 65,966.701 ±  15,719.629 | ↓ |

Figure S8: Jasmonic acid (JA) and salicylic acid (SA) responses reflect metabolic adjustment under glucose and CdCl_2_ treatments. Supernatant concentration (nmol/mL) of JA (a) and SA (c) and pellet concentrations (nmol/mg DW) of JA (b) and SA (d) from a *E. mutabilis* FAB grown in varying concentration of glucose in the presence and absence of CdCl_2_ over 5-days. Error bars represent standard deviation (*n* = 3). Statistical significance within each timepoint was assessed using a one-way ANOVA and Tukey’s HSD post-hoc test including cultures where the hormones were not detected.

**References**

1.	Kisiala A, Kambhampati S, Stock NL, Aoki M, Emery RJN. Quantification of cytokinins using high-resolution accurate-mass orbitrap mass spectrometry and parallel reaction monitoring (PRM). Anal Chem. 2019;91(23):15049–56. https://doi.org/10.1021/acs.analchem.9b03728.

2.	Nguyen NH, Nguyen QT, Dang DH, Emery RJN. Phytohormones enhance heavy metal responses in *Euglena gracilis*: Evidence from uptake of Ni, Pb and Cd and linkages to hormonomic and metabolomic dynamics. Environ Pollut. 2023;320:121094. https://doi.org/10.1016/j.envpol.2023.121094.
