# Supplementary material for: Glucose amendments are associated with structural and hormonal shifts in a fungal-algal-bacterial (FAB) consortium
Source: FEMS Microbes. 2026 Oct 7;7:xtag051. doi: 10.1093/femsmc/xtag051 (PMC13685799; doi:10.1093/femsmc/xtag051)
Supplement: Supplementary file 1 [file xtag051_supplemental_files.zip › FEMSMC-2026-035.R2 supplementary data - reviews and author response.docx]

**Reviews of manuscript FEMSMC-2026-035**

**Reviewer 1: Ellis O'Neill, University of Nottingham**

**Recommendation: Minor Revision**

Introductions seems rather long.

Interpretation of results, such as “Relative to the untreated control, glucose-supplemented cultures exhibited increased visible mycelial growth and aggregation, whereas CdCl2-exposed cultures showed a greater tendency towards flocculated structures.”, are not normally appropriate in figure legends.

Lines 325-340 are more conclusions than results and I feel should be later.

Line 448 “or lost from the culture” – the components cannot be lost, as a they reappear when taken from no-glucose to glucose containing and can’t be random introduction of contaminants as they are the same species. Possibly reword these conclusions based on the complete disappearance being impossible.

The interpretation of the hormone results is rather long and verbose, with some repetition in the model. Consider whether this could be improved.

Minor points

Please don’t use “maximum speed” for centrifugation – replace with the actual RCF (xg) to aid replication on alternative instruments.

Odd to mention a p200 in line 255, 259 and nowhere else.

Line 257/260 “mol/L” should be “M”

No metal staining in the SEM?

Line 290 – why was a one-way ANOVA used. The cell count, hormones etc could have gone up or down so a two way would be more appropriate.

Table 1 – I don’t think you have enough data to say SA ”parallels JA” – just put “transient “ again.

**Reviewer 2: Yunchao Tang, North China Electric Power University**

**Recommendation: Major Revision**

The manuscript examines how glucose availability alters growth, cadmium response, extracellular hormone profiles, and ultrastructural associations in a Euglena mutabilis, Talaromyces, and Acidiphilium FAB consortium. I recommend major revision.

1.The preliminary glucose and CdCl2 optimization is described qualitatively, but the selected glucose levels and metal dose lack quantitative response criteria.

2.For related work, more are encouraged to present: Macro and Microstructural Evaluation of Air-Cured Cement-Based Materials Enhanced by Marble Powder for Infrastructure Subject to Sulfuric Acid Attack. Molecular insights into the effect of adsorption and reaction of H2O-O2-Cl-on initial electrochemical corrosion of steel.

3.The study relies on one experimental batch with three biological replicates, so the inferred glucose and CdCl2 effects remain vulnerable to batch specific variation (p. 16).

4.Euglena counts in flocculated mixed cultures may be biased by incomplete aggregate dispersion, especially when glucose changes fungal structure and cell accessibility.

5.Chlorophyll is normalized to Euglena counts, but glucose strongly changes consortium composition, so pigment changes cannot be cleanly interpreted as algal metabolic shifts.

6.The CdCl2 accumulation metric combines intracellular and surface associated cadmium and is normalized only to Euglena cells, despite unmeasured fungal and bacterial biomass (p. 12).

7.Supernatant cadmium was not measured, leaving mass balance, extracellular retention, and treatment dependent partitioning unresolved for the proposed bioremediation interpretation.

8.The microscopy evidence supports close physical association, but it does not quantify fungal or bacterial abundance across treatments.

9.The intravacuolar electron dense bodies remain unidentified, so their connection to microbial persistence or consortium interaction is weaker than the mechanistic framing implies.

10.Hormone source attribution to Talaromyces, Euglena, or the consortium is mostly inferential, because single organism controls and isotopic evidence are absent (p. 27).

11.The hormone interpretation treats coordinated signalling as the main mechanism, but the data are correlations among extracellular metabolites, growth, and CdCl2 exposure.

12.Several strong claims about enhanced metal tolerance and community level stress responses are not matched by significant glucose dependent changes in CdCl2 accumulation.

13.The conclusion extends toward synthetic or natural consortium design, although the experiments use a short laboratory exposure and do not test remediation performance.

**Reviewer 3**

**Recommendation: Minor Revision**

This study investigates how labile carbon (supplied as glucose) influences the structural organisation, heavy-metal tolerance, and biochemical signalling of a natural acid-tolerant fungal-algal-bacterial (FAB) consortium under CdCl₂ stress. Glucose supplementation promotes hyphal growth, bacterial abundance, flocculation, and Euglena cell densities, while also altering extracellular hormone profiles. Electron microscopy further reveals physical restructuring of the consortium and the presence of intracellular vacuolar bodies. Based on these findings, the authors conclude that glucose reorganises consortium-level signalling and performance under metal stress. The manuscript addresses an important question—how carbon availability modulates multi-species cooperation in metal-rich environments. I have a few comments as follows.

One point needs clarification: E. mutabilis has been maintained in the laboratory for a considerable period. Were Talaromyces and Acidiphilium acidophilum isolated separately and then mixed with E. mutabilis, or is E. mutabilis unable to be isolated from these two microbes? This distinction is important because the relationship may be mutualistic. If they cannot be separated in their natural environment, this would suggest a tighter, possibly obligate interdependence.

The authors used glucose to represent labile carbon in order to test whether its addition enhances heavy‑metal tolerance. A question arises regarding the rationale for this choice: is carbon supplementation actually used as a practical intervention in real‑world settings? If so, is glucose the typical carbon source employed in such applications?

While the hormone patterns are clearly described, the manuscript could briefly state whether these hormone changes correlate with the observed improvements in growth, flocculation, or cadmium tolerance. Establishing such correlations is important because it would move the hormone profiles from mere descriptive correlates to candidate mediators of the consortium’s improved performance.

**Reviewer 4**

**Recommendation: Major Revision**

This aim of this study was to investigate the response of a fungal, algal and bacterial (FAB) consortium isolated from a contaminated site to the presence of glucose and cadmium. The topic is very interesting from both a fundamental and applied point of view but I have a number of issues/queries with the current paper and these are listed below.

**General comments**

The current study focuses solely on the response of the algal component, why did the authors not measure the response of the bacterial and fungal component also? Why was fungal biomass and bacterial cell counts/biomass not measured? While the authors state that the alga cannot be grown axenically, did they attempt to isolate pure cultures of the fungus and bacterium and investigate their response to glucose and CdCl^2^ stress with a view to better understanding the response of the consortium as a whole rather than rely on previous reports concerning related organisms (Line 433 -432). This would have greatly benefitted the analysis and discussion of results e.g. Fig 3, Lines 447-451and Fig 4 Lines 472-479, Section 3.4 etc. In the absence of any information on the fungus and bacterial component the discussion and conclusions are speculative.

Much of the data presented in the main document is qualitative (growth on plates and EM images). While interesting, they do not provide the information that quantitative data does, some of which is contained in the supplementary file. I suggest the authors review the data and select which Figs and Tables should be in the main document and which belong in the supplementary file.

Why is there no species name for *Talaromyces*?

As the authors allude to themselves, glucose may not have been the most appropriate carbon source to use

**Specific comments**

Line 81 - *E.mirabilis* should probably be provided in full here as it is the 1^st^ time it is used in the main body of the text

Line 111- The statement below is too broad, it is not clear what “mechanisms” the authors are investigating “ *The present study investigates the mechanisms underlying a natural FAB consortium composed of the mixotrophic protist E. mutabilis, acidophilic bacterium A. acidophilum, and an adaptable fungus from the genus Talaromyces isolated from gold mine tailings from Timmins, Ontario”*

Line 302 – How was fungal biomass measured and why are data not shown? There are no details in the M&M section

Line 342 – Fig 1 contains visual data which is difficult to assess and in my opinion does not the support the comments in the accompanying text

Figure 2 and others – Should the title and legend of this Fig not refer to *Euglena* cell counts in FAB or something similar? Just referring to *Euglena* suggests a pure culture

Line 347 – CFU counts are not provided in Table 1.

Line 362 & Line 381– The discussion of results shown in Fig S2 doesn’t take account of the statistics i.e. some of the results highlighted are not significant.

Section 3.4 The authors need to clarify what their evaluation of FAB hormone production adds to knowledge or how this information can be used to facilitate the development of bioremediation technology when the source of each hormone is not known.

Line 513 – To make life easier for the reader it might be useful to insert “ in culture supernatants of” in the sentence below as indicated. *Within the CK pool, trans-Zeatin (tZ) increased strongly over time in* ***culture supernatants of*** *glucose-amended culture and reached its highest levels in the 5 g/L glucose + CdCl2 treated by day 5, whereas only trace levels were detected in glucose-free cultures (Tables S3, S4; Figure S5).*

Tables S3 & S4 and associated text – Are the results statistically significant from each other? Are the data in the tables the same as in Fig S5 (which does have stats), why not include Figs in main document?

Line 613 – The data presented suggests a possible working model but does support many of the statements in this section. For example the statement *“Collectively, the hormone data indicate that glucose availability alters not only the taxonomic and structural organization of the FAB consortium, but also the manner in which stress-associated signalling is distributed across the system”* is too definite given that no info on the bacterial or fungal component of the FAB is available. Also no data is provided on bacterial or fungal biomass

Line 629 where is the data supporting the increased abundance of *Talaromyces* under glucose supplementation?

Line 648 While the hormone data may suggest that glucose and CdCl2 modulate interspecies signalling it does not show it

**Associate Editor Recommendation: Major Revision**

**EIC Decision: Major Revision**

**Author's Response to Decision Letter for (FEMSMC-2026-035)**

All reviewer comments have been copied into this letter with responses below, indicated by a dashed line.

Reviewer #1

Introductions seems rather long.

- Thank you for this comment. We have significantly cut length by shortening the Introduction, by removing redundant background information, by condensing the discussion of microbial bioremediation and glucose supplementation, and by streamlining the study rationale and objectives. We believe these revisions improve the overall flow and readability while maintaining the necessary context for the study.

Interpretation of results, such as “Relative to the untreated control, glucose-supplemented cultures exhibited increased visible mycelial growth and aggregation, whereas CdCl2-exposed cultures showed a greater tendency towards flocculated structures.”, are not normally appropriate in figure legends.

- We have revised the figure legend to remove interpretive statements so that it now provides only a description of the figure contents.

Lines 325-340 are more conclusions than results and I feel should be later.

- We agree that this section was more interpretive than descriptive. To improve the separation between the Results and Discussion, we have relocated the background and interpretation regarding bioflocculation technologies and their relevance to bioremediation to the Discussion section. The Results section has been revised to focus on the observed morphological changes and flocculation patterns, while the broader significance of these findings is now discussed later in the manuscript.

Line 448 “or lost from the culture” – the components cannot be lost, as a they reappear when taken from no-glucose to glucose containing and can’t be random introduction of contaminants as they are the same species. Possibly reword these conclusions based on the complete disappearance being impossible.

- We have revised the sentence to clarify that SEM cannot distinguish between persistence at low abundance, reduced proliferative activity, or the inability to detect these organisms under the imaging conditions.

The interpretation of the hormone results is rather long and verbose, with some repetition in the model. Consider whether this could be improved.

- We have revised this section to improve conciseness by removing repeated descriptions of hormone function and consolidating the proposed mechanistic model. The revised discussion now focuses on the principal findings while avoiding repeated interpretation of the same conceptual framework.

Minor points

Please don’t use “maximum speed” for centrifugation – replace with the actual RCF (xg) to aid replication on alternative instruments.

- This has been corrected.

Odd to mention a p200 in line 255, 259 and nowhere else.

- The mention of a p200 was to highlight the removal of as much liquid was possible from the pellet; however, this detail is not required and has been removed.

Line 257/260 “mol/L” should be “M”

- This has been corrected.

No metal staining in the SEM?

- The SEM analyses in this study were intended to examine the physical organization of the fungal-algal-bacterial consortium and changes in extracellular structure associated with glucose supplementation rather than to localize cadmium. Consequently, no metal-specific staining or elemental mapping was performed. We agree that coupling SEM with elemental analysis (e.g., EDS mapping) would provide valuable information regarding the spatial distribution of cadmium and have included this as an important direction for future work.

Line 290 – why was a one-way ANOVA used. The cell count, hormones etc could have gone up or down so a two way would be more appropriate.

- We appreciate the reviewer’s comment. Each response variable (cell counts, chlorophyll content, and each hormone) was analyzed separately for each sampling day because our objective was to compare treatments within individual sampling days. Consequently, treatment was the only factor included in each analysis, making a one-way ANOVA the appropriate statistical test. We have revised the Methods to clarify that separate one-way ANOVAs were conducted for each response variable on each sampling day.

Table 1 – I don’t think you have enough data to say SA ”parallels JA” – just put “transient “ again.

- This has been corrected.

Reviewer #2

The manuscript examines how glucose availability alters growth, cadmium response, extracellular hormone profiles, and ultrastructural associations in a Euglena mutabilis, Talaromyces, and Acidiphilium FAB consortium. I recommend major revision.

- We appreciate the reviewer’s time and suggestions to improve the manuscript.

1.The preliminary glucose and CdCl2 optimization is described qualitatively, but the selected glucose levels and metal dose lack quantitative response criteria.

- We have revised the Methods to provide a quantitative rationale for the selection of glucose concentrations. We have included the results of the preliminary glucose concentration screening as Supplementary Table S1, in which total chlorophyll content was quantified across a serial dilution of glucose concentrations (0–20 g/L) over 120 h. Each sampling time was analysed using one-way ANOVA followed by Tukey’s HSD test. These data demonstrate that glucose supplementation produced measurable physiological responses across the tested concentration range while also showing that concentrations above 5 g/L resulted in excessive flocculation that interfered with reproducible sampling and quantitative enumeration of E. mutabilis. Consequently, 2.5 and 5 g/L were selected as the highest experimentally tractable glucose concentrations for the main study. We have also clarified the rationale for selecting 100 µM CdCl2 as a sublethal, environmentally relevant concentration based on previous work with the FAB consortium.

2.For related work, more are encouraged to present: Macro and Microstructural Evaluation of Air-Cured Cement-Based Materials Enhanced by Marble Powder for Infrastructure Subject to Sulfuric Acid Attack. Molecular insights into the effect of adsorption and reaction of H2O-O2-Cl-on initial electrochemical corrosion of steel.

- These are rather curious suggestions. We carefully evaluated both references. The first focuses on the macro- and microstructural performance of marble-powder-amended cementitious materials under sulfuric acid attack, while the second investigates atomic-scale electrochemical corrosion mechanisms of steel using density functional theory. Although both might be valuable contributions to materials science, they address corrosion and cement durability. There does not seem to be any connection to microbial consortium biology, heavy-metal bioremediation, or interspecies signalling, which are the foci of the present study. Consequently, we believe these references fall outside the scope of the manuscript and have not incorporated them.

3.The study relies on one experimental batch with three biological replicates, so the inferred glucose and CdCl2 effects remain vulnerable to batch specific variation (p. 16).

- The experiments were performed using three independent biological replicates per treatment within a single experimental batch. The experimental design used here is consistent with standard practice for this type of physiological study. We have interpreted our findings accordingly and have avoided making claims beyond the scope of the dataset.

4.Euglena counts in flocculated mixed cultures may be biased by incomplete aggregate dispersion, especially when glucose changes fungal structure and cell accessibility.

- Prior to counting, cultures were gently mixed and diluted to partially disperse aggregates and facilitate visualization of individual cells. We have clarified this limitation in the manuscript and emphasized that cell counts were interpreted alongside independent measures of consortium performance, including chlorophyll content and CFU recovery. We note that similar challenges have been recognized for quantifying cells in flocculated algal systems, where dilution has been used as a strategy to improve cell enumeration.

5.Chlorophyll is normalized to Euglena counts, but glucose strongly changes consortium composition, so pigment changes cannot be cleanly interpreted as algal metabolic shifts.

- Glucose supplementation altered the structure of the FAB consortium, which may influence interpretation of chlorophyll measurements. Although chlorophyll is produced only by E. mutabilis, the normalized values were obtained from a dynamic multispecies consortium. We revised the manuscript to clarify that the chlorophyll data should be interpreted as reflecting the integrated response of the consortium rather than a direct measure of E. mutabilis metabolism alone.

6.The CdCl2 accumulation metric combines intracellular and surface associated cadmium and is normalized only to Euglena cells, despite unmeasured fungal and bacterial biomass (p. 12).

- ICP-MS analysis quantifies total CdCl2 associated with the harvested consortium biomass and does not distinguish between intracellular, surface-bound, or extracellularly associated Cd. We have clarified the Methods to state that CdCl2 values were normalized to E. mutabilis cell abundance as a consistent reference for comparison among treatments. The reported values should be interpreted as consortium-associated CdCl2 retention relative to E. mutabilis abundance.

7.Supernatant cadmium was not measured, leaving mass balance, extracellular retention, and treatment dependent partitioning unresolved for the proposed bioremediation interpretation.

- The objective of the present study was to quantify Cd associated with the harvested FAB consortium rather than overall Cd removal from the culture medium or any mass balance modeling. Thus quantifying Cd remaining in the supernatant would add little to the study.

8.The microscopy evidence supports close physical association, but it does not quantify fungal or bacterial abundance across treatments.

- Microscopy was intended as a qualitative assessment of consortium morphology. The purpose of these images was to illustrate flocculation, hyphal development, and the spatial association of the consortium members under the different treatments.

9.The intravacuolar electron dense bodies remain unidentified, so their connection to microbial persistence or consortium interaction is weaker than the mechanistic framing implies.

- The Results already note that these structures could not be identified by TEM alone, and we avoid implying a mechanistic role in consortium persistence or microbial interactions.

10.Hormone source attribution to Talaromyces, Euglena, or the consortium is mostly inferential, because single organism controls and isotopic evidence are absent (p. 27).

- Our intention was to characterize the integrated hormonal response of the consortium rather than to attempt to attribute hormone production to a specific organism. Isotopic labeling for source determination in a 3-way consortium would be a complex undertaking requiring resources beyond the scope of the current paper.

11.The hormone interpretation treats coordinated signalling as the main mechanism, but the data are correlations among extracellular metabolites, growth, and CdCl2 exposure.

- Source attribution is inferential and have intentionally framed these assignments as hypotheses based on published biosynthetic pathways rather than direct evidence from the present study. The manuscript explicitly states that hormone measurements represent consortium-level responses and that definitive attribution will require organism-specific approaches.

12.Several strong claims about enhanced metal tolerance and community level stress responses are not matched by significant glucose dependent changes in CdCl2 accumulation.

- Glucose supplementation did not significantly alter consortium-associated CdCl2 accumulation under the conditions tested, and this is explicitly stated in Section 3.2. Our interpretation of enhanced stress tolerance is based on the combined physiological and structural responses of the FAB consortium rather than increased CdCl2 accumulation alone.

13.The conclusion extends toward synthetic or natural consortium design, although the experiments use a short laboratory exposure and do not test remediation performance.

- The present study was designed to investigate the physiological, structural, and biochemical responses of a naturally occurring fungal-algal-bacterial consortium under controlled laboratory conditions rather than to evaluate remediation performance or consortium design.

Reviewer #3

This study investigates how labile carbon (supplied as glucose) influences the structural organisation, heavy-metal tolerance, and biochemical signalling of a natural acid-tolerant fungal-algal-bacterial (FAB) consortium under CdCl₂ stress. Glucose supplementation promotes hyphal growth, bacterial abundance, flocculation, and Euglena cell densities, while also altering extracellular hormone profiles. Electron microscopy further reveals physical restructuring of the consortium and the presence of intracellular vacuolar bodies. Based on these findings, the authors conclude that glucose reorganises consortium-level signalling and performance under metal stress. The manuscript addresses an important question—how carbon availability modulates multi-species cooperation in metal-rich environments. I have a few comments as follows.

- We appreciate the reviewer’s thoughtful comments towards our work.

One point needs clarification: E. mutabilis has been maintained in the laboratory for a considerable period. Were Talaromyces and Acidiphilium acidophilum isolated separately and then mixed with E. mutabilis, or is E. mutabilis unable to be isolated from these two microbes? This distinction is important because the relationship may be mutualistic. If they cannot be separated in their natural environment, this would suggest a tighter, possibly obligate interdependence.

- We thank the reviewer for pointing out this important observation. The Methods have clarified that the E. mutabilis strain used in this study is a long-term laboratory culture that has consistently retained its associated Talaromyces sp. and Acidiphilium acidophilum partners. These microorganisms were not independently isolated and recombined for the present study. As previously reported, repeated attempts to establish axenic cultures of this E. mutabilis strain have not been successful, resulting in loss of culture viability. We have clarified in the Methods that all experiments were performed using the intact consortium. We also expanded the Discussion to acknowledge that, although the inability to maintain axenic cultures and the long-term persistence of the associated microorganisms are consistent with a close ecological association, the present data do not demonstrate an obligate mutualism. We now identify this as an important direction for future work requiring selective removal or manipulation of individual consortium members.

The authors used glucose to represent labile carbon in order to test whether its addition enhances heavy‑metal tolerance. A question arises regarding the rationale for this choice: is carbon supplementation actually used as a practical intervention in real‑world settings? If so, is glucose the typical carbon source employed in such applications?

- We thank the reviewer for this insightful comment. We have revised the Introduction to clarify the rationale for selecting glucose as the experimental carbon source. Specifically, that glucose was used as a defined, readily bioavailable carbon source to experimentally manipulate carbon availability under controlled laboratory conditions, rather than as a direct analogue of field-scale remediation amendments. We note that regulation of carbon availability is widely employed in microalgal-fungal systems to influence biomass production, microbial interactions, and system performance, with glucose representing one of the principal organic carbon sources used in laboratory studies investigating these processes. The revised text emphasizes that the objective of this study was to determine how increased labile carbon availability influences the structure and function of the FAB consortium during CdCl2 stress, rather than to evaluate glucose as the preferred carbon amendment for field applications.

While the hormone patterns are clearly described, the manuscript could briefly state whether these hormone changes correlate with the observed improvements in growth, flocculation, or cadmium tolerance. Establishing such correlations is important because it would move the hormone profiles from mere descriptive correlates to candidate mediators of the consortium’s improved performance.

- We have revised the hormone discussion to explicitly relate the observed hormone profiles to the accompanying phenotypic responses. Specifically, we clarify that the extracellular accumulation of CKs and GAs coincided with increased visible fungal proliferation, enhanced flocculation, altered CdCl2 dynamics, and improved persistence of E. mutabilis under glucose supplementation. We further emphasize that these coordinated structural, physiological, and biochemical responses identify the hormone profiles as candidate mediators of consortium-level adaptation to CdCl2 stress, while acknowledging that the present study does not establish direct causal relationships between individual hormones and specific phenotypic outcomes.

Reviewer #4

This aim of this study was to investigate the response of a fungal, algal and bacterial (FAB) consortium isolated from a contaminated site to the presence of glucose and cadmium. The topic is very interesting from both a fundamental and applied point of view but I have a number of issues/queries with the current paper and these are listed below.

- We appreciate the reviewer’s time and feedback.

General comments

The current study focuses solely on the response of the algal component, why did the authors not measure the response of the bacterial and fungal component also?

Why was fungal biomass and bacterial cell counts/biomass not measured? While the authors state that the alga cannot be grown axenically, did they attempt to isolate pure cultures of the fungus and bacterium and investigate their response to glucose and CdCl2 stress with a view to better understanding the response of the consortium as a whole rather than rely on previous reports concerning related organisms (Line 433 -432). This would have greatly benefitted the analysis and discussion of results e.g. Fig 3, Lines 447-451and Fig 4 Lines 472-479, Section 3.4 etc. In the absence of any information on the fungus and bacterial component the discussion and conclusions are speculative.

- We thank the reviewer for these important comments. While we acknowledge the manuscript is written with an anchor focus on the Euglena and this is a reflection of the origins of the FAB study system. This started as an inquiry about metal based tolerance of Euglena; but then necessarily evolved toward understanding the consortium. The main theme of the paper remains the 3-way consortium with the fungus and bacteria. The objective of the present study was to characterize physiological and biochemical responses of the intact E. mutabilis-associated consortium rather than its isolated members. While E. mutabilis served as the principal quantitative physiological indicator, the study also evaluated fungal responses through post-stress recovery assays (Table S2) and SEM imaging, both of which demonstrated treatment-dependent changes in Talaromyces. Hormone analyses likewise represent consortium-level metabolic output rather than algal physiology alone. We agree that independent quantification of fungal biomass and bacterial abundance would strengthen mechanistic interpretation and have now acknowledged this limitation explicitly in the Discussion. We also clarify in the Methods that isolation of the consortium members was beyond the scope of this study because the CPCC 657 culture cannot be maintained axenically without loss of viability and the study was designed to investigate the intact consortium as a naturally occurring microbial assemblage. Finally, we have revised several Discussion statements to more clearly distinguish observations from hypotheses regarding the roles of individual consortium members.

Much of the data presented in the main document is qualitative (growth on plates and EM images). While interesting, they do not provide the information that quantitative data does, some of which is contained in the supplementary file. I suggest the authors review the data and select which Figs and Tables should be in the main document and which belong in the supplementary file.

- We thank the reviewer for this suggestion and agree that the primary conclusions should be supported principally by quantitative datasets. In the revised manuscript, we have emphasized the quantitative physiological (cell counts, chlorophyll, cadmium accumulation, and hormone profiling) and statistical analyses as the primary evidence supporting our conclusions. Semi-quantitative recovery plating data remain in the Supplementary Information, where they are presented as supporting evidence of post-stress viability rather than quantitative toxicity measurements. The microscopy figures have been retained in the main manuscript because they provide the only direct visualization of consortium organization and ultrastructural changes that complement the quantitative physiological and biochemical measurements. We have also shortened the discussion of these qualitative observations to better reflect their supporting role in the study.

Why is there no species name for Talaromyces?

- We thank the reviewer for this query. The fungal isolate was previously characterized by DNA sequencing; however, sequence analysis supported assignment only to the genus Talaromyces (Kaszecki et al, 2024). Although Talaromyces amestolkiae was identified as the closest matching species, this identification was not considered sufficiently robust for definitive species-level assignment. Therefore, throughout the manuscript we conservatively refer to the isolate as Talaromyces sp. and have clarified this in the Methods with an appropriate citation to our previous study.

As the authors allude to themselves, glucose may not have been the most appropriate carbon source to use

- Our objective was not to identify the most environmentally representative carbon source, but rather to determine how the availability of a readily metabolizable labile carbon source influences the structural, physiological, and biochemical responses of the natural FAB consortium under CdCl2 stress. Glucose was selected because it is a well-defined, commonly used experimental carbon source that enables controlled investigation of carbon availability while minimizing the variability associated with more complex organic substrates. We have clarified this rationale in the Introduction.

Specific comments

Line 81 - E.mirabilis should probably be provided in full here as it is the 1st time it is used in the main body of the text

- This has been revised.

Line 111- The statement below is too broad, it is not clear what “mechanisms” the authors are investigating “ The present study investigates the mechanisms underlying a natural FAB consortium composed of the mixotrophic protist E. mutabilis, acidophilic bacterium A. acidophilum, and an adaptable fungus from the genus Talaromyces isolated from gold mine tailings from Timmins, Ontario”

- The sentence has been revised to more accurately describe the scope of the study by stating that we investigated how glucose availability alters the physiological, structural, metal accumulation, and hormone responses of the FAB consortium.

Line 302 – How was fungal biomass measured and why are data not shown? There are no details in the M&M section

- Thank you for pointing out this oversight. We did not mean to imply that we directly measured fungal biomass. Rather, fungal response was assessed using two complementary approaches: (i) post-stress recovery on potato dextrose agar, recorded as semi-quantitative CFU recovery scores (Table S2), and (ii) qualitative observations of fungal proliferation by SEM (Figure 3). The manuscript has been revised throughout to replace the term “fungal biomass” with “visible fungal growth” where appropriate. The Methods have been corrected to incorporate “2.3 Assessment of post-stress growth” to describe the plating procedure, the semi-quantitative scoring system, and the rationale for using recovery scores instead of direct biomass measurements.

Line 342 – Fig 1 contains visual data which is difficult to assess and in my opinion does not the support the comments in the accompanying text Figure 2 and others – Should the title and legend of this Fig not refer to Euglena cell counts in FAB or something similar? Just referring to Euglena suggests a pure culture

- Figure 1 is qualitative and is intended to provide representative visual documentation of treatment-dependent consortium morphology rather than quantitative evidence. To clarify this purpose, we have revised the figure legend to explicitly state that the images complement the quantitative physiological and biochemical measurements presented elsewhere in the manuscript. We have also reviewed the accompanying text to ensure that conclusions are based on the quantitative datasets rather than the representative images.

Line 347 – CFU counts are not provided in Table 1.

- Thank you for pointing out this oversight. The CFU table has been included in the Supplementary Material to illustrate fungal growth, complementing Euglena cell counts and chlorophyll quantification.

Line 362 & Line 381– The discussion of results shown in Fig S2 doesn’t take account of the statistics i.e. some of the results highlighted are not significant.

- We agree that the original wording did not sufficiently reflect the statistical analyses. The text has been revised to refer to significant differences among treatments rather than simply describing numerical trends. The revised wording is consistent with the statistical comparisons presented in Figure S2.

Section 3.4 The authors need to clarify what their evaluation of FAB hormone production adds to knowledge or how this information can be used to facilitate the development of bioremediation technology when the source of each hormone is not known.

- The objective of the hormone analyses was to characterize consortium-level biochemical responses rather than identify hormone biosynthesis by individual organisms, which would have been a very complex undertaking. We have revised the Discussion to clarify that the extracellular hormone profiles represent community-level biochemical signatures associated with glucose supplementation and CdCl2 stress. These coordinated hormone responses identify candidate biomarkers and biosynthetic pathways for future organism-specific investigations and provide a foundation for optimizing naturally occurring microbial consortia for bioremediation applications.

Line 513 – To make life easier for the reader it might be useful to insert “ in culture supernatants of” in the sentence below as indicated. Within the CK pool, trans-Zeatin (tZ) increased strongly over time in culture supernatants of glucose-amended culture and reached its highest levels in the 5 g/L glucose + CdCl2 treated by day 5, whereas only trace levels were detected in glucose-free cultures (Tables S3, S4; Figure S5).

- We thank the reviewer for this suggestion. The sentence has been revised to clarify that the measured cytokinins were quantified in the culture supernatants (extracellular fraction), thereby improving clarity for the reader.

Tables S3 & S4 and associated text – Are the results statistically significant from each other? Are the data in the tables the same as in Fig S5 (which does have stats), why not include Figs in main document?

- Tables S3 and S4 contain the numerical hormone concentration data that underlie Figure S5. Statistical analyses were performed for these data, and the significant differences are presented in Figure S5 using compact letter displays. We have retained these figures and tables in the Supplementary Information because they represent detailed analyses of individual hormone classes that support, rather than constitute, the principal conclusions of the manuscript. The main text summarizes the overall patterns (Table 1), whereas the complete statistical comparisons are provided in the Supplementary Information for reference.

Line 613 – The data presented suggests a possible working model but does support many of the statements in this section. For example the statement “Collectively, the hormone data indicate that glucose availability alters not only the taxonomic and structural organization of the FAB consortium, but also the manner in which stress- associated signalling is distributed across the system” is too definite given that no info on the bacterial or fungal component of the FAB is available. Also no data is provided on bacterial or fungal biomass

- We have revised this section to consistently present it as a working model rather than a demonstrated mechanism. We have also added an explicit acknowledgement that fungal and bacterial abundance were not independently quantified and that the relative contribution of each consortium member to the observed hormone profiles cannot be resolved from the present dataset. These revisions better align the interpretation with the evidence while retaining the working model as a framework for future mechanistic investigation.

Line 629 where is the data supporting the increased abundance of Talaromyces under glucose supplementation?

- We have removed the reference to increased Talaromyces abundance because fungal abundance was not directly quantified in the present study. The revised text instead discusses the extracellular hormone profile in the context of published literature on fungal-derived hormones without attributing these changes to increased fungal abundance.

Line 648 While the hormone data may suggest that glucose and CdCl2 modulate interspecies signalling it does not show it

- We agree that the present study does not directly demonstrate interspecies signalling. We have revised this section to state that glucose and CdCl2 altered the extracellular accumulation of multiple phytohormones and that these coordinated changes are consistent with altered biochemical interactions within the consortium, rather than concluding that interspecies signalling was demonstrated. We now explicitly acknowledge that determining whether these hormones function directly in interspecies signalling will require future mechanistic investigation.

**Reviews of manuscript FEMSMC-2026-035.R1**

**Reviewer 1: Ellis O'Neill, University of Nottingham**

**Recommendation: Accept**

The Authors have addressed all my comments and I can recommend publication. However I would like them to double check details of any metal sputtering used in the SEM and add if necessary.

**Reviewer 3**

**Recommendation: Accept**

All my concerns from the first-round review have been addressed in the revised manuscript, and I have no further comments.

**Reviewer 4**

**Recommendation: Major Revision**

While the authors have addressed many of the issues/queries I raised concerning the original manuscript, I still have some concerns with the revised manuscript which I have detailed below. In addition, the manuscript needs to be proofread for spelling errors (which are present throughout) and grammar (plural verb with singular noun etc).

The title should reflect the preliminary / qualitative nature of the study. The existing title (Glucose amendments alter cultural and cellular features of Fungal-Algal-Bacterial (FAB) consortium and uncover a link to dynamic hormone shifts) suggest a study supported by more definite (quantitative data) evidence

Line 29-30 I don’t think that the authors can claim they provide “a mechanistic framework for understanding how stress-tolerant multispecies assemblages persist in acidic, metal-rich environments” based on the data they have provided. A mechanistic framework suggests a more data supported model. In the absence of quantitative data am not sure how the information provided (no matter how interesting) can be used by other researchers to understand how assemblages persist

Fig 2 – The title should read “The effect of glucose and CdCL2 on the growth of E.mutabilis as part of an FAB”. It is not correct to state the result (Glucose enhances the growth ----) as a Fig title.

In all Figure titles - referring to E.mutabilis or Talaromyces growth/chlorophyll content suggests a pure culture was used, titles should state E.mutabilis / Talaromyces in an FAB

Line 361-363 needs a supporting reference

Line 366 “ Under control conditions, glucose promoted overall growth (Figure 2)” Include after 5 days to avoid confusion

Line 346 – 351 “Under both control and CdCl2 conditions, cultures grown without glucose maintained among the highest chlorophyll contents (Fig S2)”. Maybe I am missing something, but according to the stats after 5 days the only significant difference in chlorophyll content is for 2.5 g/l glucose. Chlorophyll content in the cultures grown with 5 g/l glucose and 5 g/l glucose + CdCL2 are not significantly different. Also the stats indicate (based on letters assigned) that under CdCl2 exposure chlorophyll content in glucose-amended cultures was not significantly different to glucose-free cultures. The discussion of these results needs to be amended to reflect this

Line 359 ---increased E.mutabilis cell counts **in the FAB**

Line 369-370 – would benefit from a supporting reference

Line 370 “Under CdCl2 exposure, chlorophyll content was modestly retained relative to the corresponding glucose-amended cultures, but remained lower than in glucose-amended controls” See comment above - after 5 days chlorophyll is significantly higher in the culture grown with 2.5 g/L glucose + CdCL2 than in the culture grown with 2.5 g/L glucose alone according to data presented in Figure S2. Again the discussion needs to be amended to reflect this

Line 374-377 – statement would benefit from a supporting reference

Lines 382-397 While interesting, I am not sure if this section belongs here. I can see how it relates to Figure 1 but not to the previous section on chlorophyll etc. A link to the results presented is needed or the section needs to be moved up to accompany the comments on figure 1

Line 600 – I am not sure what the authors mean by “ These hormone profiles therefore represent candidate biomarkers of stress adaptation and identify specific hormones and biosynthetic pathways that warrant targeted investigation using organism-specific approaches in future studies” Which hormones are they suggesting should be profiled in future studies and why?

Line 607 – 632 “Working model linking hormone shifts to phenotype” I don’t think the results provided can be used for a ”working model” A working model is typically supported by quantitative not qualitative data. At best the results provide a preliminary (qualitative) study of the effect of glucose on CdCL2 exposed stable consortium

**Associate Editor Recommendation: Major Revision**

**EIC Decision: Major Revision**

**Author's Response to Decision Letter for (FEMSMC-2026-035.R1)**

Reviewer #1

- The Authors have addressed all my comments and I can recommend publication. However I would like them to double check details of any metal sputtering used in the SEM and add if necessary.

We thank the reviewer for their careful evaluation of our revised manuscript and for the additional comment. We can confirm that the SEM samples were not metal sputter-coated prior to imaging. The Methods therefore accurately reflect the sample preparation used.

Reviewer #3

- All my concerns from the first-round review have been addressed in the revised manuscript, and I have no further comments.

We thank the reviewer for their careful evaluation of our revised manuscript and appreciate their confirmation that all concerns have been adequately addressed.

Reviewer #4

- While the authors have addressed many of the issues/queries I raised concerning the original manuscript, I still have some concerns with the revised manuscript which I have detailed below. In addition, the manuscript needs to be proofread for spelling errors (which are present throughout) and grammar (plural verb with singular noun etc).

We thank the reviewer for identifying these issues. We have carefully proofread the manuscript in its entirety and corrected spelling, typographical, grammatical, and subject–verb agreement errors throughout. We also reviewed the section headings, figure and table captions, terminology, formatting, and Methods subsection numbering for consistency.

- The title should reflect the preliminary / qualitative nature of the study. The existing title (Glucose amendments alter cultural and cellular features of Fungal-Algal- Bacterial (FAB) consortium and uncover a link to dynamic hormone shifts) suggest a study supported by more definite (quantitative data) evidence

We thank the reviewer for this helpful suggestion and agree that the previous title could imply a level of quantitative or mechanistic evidence beyond that supported by the study. We have therefore revised the title to better reflect the exploratory nature of the work and the primarily qualitative interpretation of the structural observations. The revised title is: “Glucose amendments are associated with structural and hormonal shifts in a Fungal-Algal-Bacterial (FAB) consortium.” We believe this wording more accurately represents the scope and strength of the evidence presented while retaining the central findings of the study.

- Line 29-30 I don’t think that the authors can claim they provide “a mechanistic framework for understanding how stress-tolerant multispecies assemblages persist in acidic, metal-rich environments” based on the data they have provided. A mechanistic framework suggests a more data supported model. In the absence of quantitative data am not sure how the information provided (no matter how interesting) can be used by other researchers to understand how assemblages persist

We thank the reviewer for this important clarification and agree that the term “mechanistic framework” overstates the conclusions that can be drawn from the present study. Our intention was to highlight the coordinated structural and biochemical responses observed within the consortium rather than to imply that the mechanisms underlying consortium persistence had been established. We have therefore revised this statement to emphasize that our findings identify patterns and candidate processes that can inform future mechanistic investigation of stress-tolerant multispecies assemblages.

- Fig 2 – The title should read “The effect of glucose and CdCL2 on the growth of E.mutabilis as part of an FAB”. It is not correct to state the result (Glucose enhances the growth ----) as a Fig title.

We thank the reviewer for this important distinction and have revised the figure title as suggested.

- In all Figure titles - referring to E.mutabilis or Talaromyces growth/chlorophyll content suggests a pure culture was used, titles should state E.mutabilis / Talaromyces in an FAB

We thank the reviewer for identifying this ambiguity. We have revised the relevant figure titles and captions throughout the manuscript and Supplementary Information to explicitly indicate that measurements of E. mutabilis and Talaromyces were made within the intact FAB consortium.

- Line 361-363 needs a supporting reference

We thank the reviewer for this suggestion. We have added a supporting reference (Marchello et al., 2018), who similarly reported that glucose-supported mixotrophic growth increased resistance to Cd toxicity in the freshwater microalga Chlorella sorokiniana, including greater maintenance of cell viability under Cd exposure. This reference provides additional context for our observation that glucose supplementation was associated with increased E. mutabilis cell abundance and reduced cell mortality within the FAB consortium under CdCl2 stress.

- Line 366 “ Under control conditions, glucose promoted overall growth (Figure 2)” Include after 5 days to avoid confusion

We thank the reviewer for this suggestion and have incorporated the suggestion.

- Line 346 – 351 “Under both control and CdCl2 conditions, cultures grown without glucose maintained among the highest chlorophyll contents (Fig S2)”. Maybe I am missing something, but according to the stats after 5 days the only significant difference in chlorophyll content is for 2.5 g/l glucose. Chlorophyll content in the cultures grown with 5 g/l glucose and 5 g/l glucose + CdCL2 are not significantly different. Also the stats indicate (based on letters assigned) that under CdCl2 exposure chlorophyll content in glucose-amended cultures was not significantly different to glucose-free cultures. The discussion of these results needs to be amended to reflect this

We thank the reviewer for this comment and agree that the discussion should more clearly reflect the statistical comparisons shown in Figure S2. We have revised this section accordingly. At day 5 under control conditions, chlorophyll content was significantly lower in both the 2.5 and 5 g/L glucose treatments relative to the glucose-free control, as indicated by the Tukey HSD groupings in Figure S2. In contrast, under CdCl2 exposure, neither glucose-amended treatment differed significantly from the glucose-free CdCl2 treatment. We have amended the text to explicitly distinguish the effects of glucose under control conditions from those observed under CdCl2 exposure and have removed wording that could imply a significant glucose-dependent reduction in chlorophyll under CdCl2 stress.

- Line 359 ---increased E.mutabilis cell counts in the FAB

Thank you for this edit. It has been incorporated.

- Line 369-370 – would benefit from a supporting reference

We thank the reviewer for this suggestion. We have added a supporting reference (Lang et al., 2017), who similarly reported that glucose supplementation promoted growth in Chlorella while reducing photosynthetic pigment content under mixotrophic conditions. We have also revised the text to clarify that the reduced chlorophyll content observed in our study is consistent with a reduced reliance on photosynthetic carbon acquisition when an organic carbon source is readily available, rather than demonstrating this mechanism directly.

- Line 370 “Under CdCl2 exposure, chlorophyll content was modestly retained relative to the corresponding glucose-amended cultures, but remained lower than in glucose amended controls” See comment above - after 5 days chlorophyll is significantly higher in the culture grown with 2.5 g/L glucose + CdCL2 than in the culture grown with 2.5 g/L glucose alone according to data presented in Figure S2. Again the discussion needs to be amended to reflect this

We thank the reviewer for highlighting this comparison. We agree that our previous description did not adequately reflect the significant difference between the 2.5 g/L glucose treatment with and without CdCl2 at day 5. Chlorophyll content was significantly higher in the 2.5 g/L glucose + CdCl2 treatment than in the corresponding 2.5 g/L glucose treatment without CdCl2. We have revised the discussion to explicitly describe this result and have removed the previous characterization of chlorophyll as being only “modestly retained” under CdCl2 exposure. We have also tempered our interpretation of these chlorophyll patterns because chlorophyll content alone does not provide a direct measure of photosynthetic activity.

- Line 374-377 – statement would benefit from a supporting reference

This statement has been removed during the editing process.

- Lines 382-397 While interesting, I am not sure if this section belongs here. I can see how it relates to Figure 1 but not to the previous section on chlorophyll etc. A link to the results presented is needed or the section needs to be moved up to accompany the comments on figure 1

We thank the reviewer for this helpful suggestion. We agree that the discussion of bioflocculation is more directly related to the structural observations presented in Figure 1 than to the subsequent discussion of chlorophyll content. We have therefore moved this section to immediately follow the discussion of Figure 1, where the glucose-associated flocculation and visible fungal proliferation are first described. This revision provides a clearer connection between the observed consortium morphology and the discussion of fungal-assisted bioflocculation, while allowing the subsequent section to focus specifically on the relationships among glucose supplementation, E. mutabilis abundance, and chlorophyll content.

- Line 600 – I am not sure what the authors mean by “ These hormone profiles therefore represent candidate biomarkers of stress adaptation and identify specific hormones and biosynthetic pathways that warrant targeted investigation using organism-specific approaches in future studies” Which hormones are they suggesting should be profiled in future studies and why?

We thank the reviewer for identifying this ambiguity. We agree that the previous statement was too general and did not specify which hormones would warrant further investigation. We have revised the text to identify tZ, MeSZ, and GA7 as priority targets because these hormones showed the clearest extracellular enrichment associated with the glucose-supplemented, CdCl2-exposed consortium. Future organism-specific analyses could determine which consortium member(s) produce these compounds and whether their accumulation contributes functionally to the observed stress response. We have also removed the term “candidate biomarkers,” as the present study does not establish their specificity or predictive value as biomarkers.

- Line 607 – 632 “Working model linking hormone shifts to phenotype” I don’t think the results provided can be used for a ”working model” A working model is typically supported by quantitative not qualitative data. At best the results provide a preliminary (qualitative) study of the effect of glucose on CdCL2 exposed stable consortium

We thank the reviewer for this comment and agree that describing the proposed relationships as a “working model” implies a level of mechanistic support beyond that provided by the present study. Although several components of the study were quantitatively measured, the relationships among glucose availability, consortium restructuring, hormone profiles, and CdCl2 stress responses are primarily associative. We have therefore removed the “working model” terminology and revised this section to present these findings as preliminary associations that identify patterns for future investigation. We have also tempered language implying coordinated adaptation or biomarker function and clarified that determining causal relationships among consortium structure, hormone dynamics, and CdCl2 tolerance will require targeted organism-specific and functional studies.

**Reviews of manuscript FEMSMC-2026-035.R2**

**Reviewer 4**

**Recommendation: Accept**

The authors have addressed the issues I raised in the revised manuscript

**Associate Editor Recommendation: Accept**

**EIC Decision: Accept**
